# Structural basis for human DPP4 receptor recognition by MERS-like coronaviruses 2014-422 and GX2012

**DOI:** 10.1371/journal.ppat.1013792

**Published:** 2026-01-07

**Authors:** Zichun Lin, Teng Gao, Xinquan Wang

**Affiliations:** 1 Beijing Frontier Research Center for Biological Structure, Beijing, China; 2 School of Life Sciences, Tsinghua University, Beijing, China; 3 Joint Graduate Program of Peking-Tsinghua-National Institute of Biological Science, Tsinghua University, Beijing, China; Washington State University, UNITED STATES OF AMERICA

## Abstract

Since its emergence in 2012, Middle East respiratory syndrome coronavirus (MERS-CoV) has posed a significant threat to human health. Recently, novel MERS-like coronaviruses with the potential for cross-species transmission have been identified. In this study, we focused on two newly isolated bat strains with putative health concern: BatCoV/Ii/GD/2014-422 (2014-422) and BtTp-BetaCoV/GX2012 (GX2012). We determined the cryo-EM structures of the spike glycoprotein trimer in the closed state for these two viruses. These structures display a more compact conformation compared to MERS-CoV spike. Biochemical characterization demonstrates that the spike receptor-binding domains (RBDs) of 2014-422 and GX2012 can bind to human dipeptidyl peptidase 4 (hDPP4). To investigate the structural determinants of pseudovirus infection, we solved the cryo-EM structures of 2014-422 RBD-hDPP4 and GX2012 RBD-hDPP4 complexes. The binding mode of the complex is conserved, but the angle of the RBD binding undergoes significant tilting. Detailed structural analysis reveals that an additional residue at position 514 interacts with the N321 glycan in hDPP4, altering the binding angle and thus influencing receptor recognition. These findings offer valuable insights into the receptor utilization of *Merbecovirus* and provide a structural basis for future surveillance efforts.

## Author summary

Two MERS-like coronaviruses, BatCoV/Ii/GD/2014-422 (2014-422) and BtTp-BetaCoV/GX2012 (GX2012), have recently emerged as potential zoonotic threats. In this study, we provide a detailed structural analysis of these two viruses, focusing on their spike proteins and interactions with human Dipeptidyl Peptidase 4 (hDPP4), the receptor used by MERS-CoV. Using cryo-electron microscopy (cryo-EM), we determined high-resolution structures of 2014-422 and GX2012 spike glycoproteins in their closed conformations. Our results show that GX2012 mediates efficient pseudovirus entry into human cells, whereas 2014-422 shows markedly reduced entry efficiency. This difference is linked to structural variations, including a unique residue at position 514 in both viruses that affects the RBD binding angle and receptor recognition. Together, these findings provide key insights into the structural basis of receptor usage by MERS-like coronaviruses and suggest that HKU4r-CoVs may acquire the ability to infect human cells through two evolutionary routes: within bats and through pangolins as intermediate hosts.

## Introduction

Coronaviruses are a group of enveloped, positive-sense RNA viruses classified as type I membrane fusion viruses, employing spike (S) proteins to mediate viral entry into the host cell [1]. The spike protein is a homotrimer composed of two subunits: S1 and S2. The S1 subunit primarily facilitates receptor recognition and binding, with most coronaviruses utilizing the C-terminal domain (CTD) as the receptor-binding domain (RBD) [2]. While the S2 subunit is involved in the membrane fusion process [3]. Structural transitions of the RBD between “down” and “up” positions are critical for receptor engagement: when all three RBDs adopt the down conformation, the spike trimer maintains a closed conformation that sterically hinders receptor interaction. Transition to an open conformation occurs when at least one RBD adopts the up position, enabling efficient receptor binding [4,5].

The Middle East Respiratory Syndrome Coronavirus (MERS-CoV), first identified in 2012, remains a significant cause of concern due to its high case fatality rate, which has been reported to reach 36% [6]. MERS-CoV belongs to the *Merbecovirus* lineage within the *Betacoronavirus* genus and utilizes human dipeptidyl peptidase 4 (hDPP4) as its primary receptor. Although MERS-CoV is believed to have originated in bats [7,8], with dromedary camels acting as an intermediate host [9,10], the lack of a known virus exhibiting a whole-genome sequence with high homology to MERS-CoV prevents definitive conclusions about its evolutionary origins [1]. Thus, continued research is necessary to comprehensively define the zoonotic transmission dynamics and ancestral lineage of MERS-CoV.

In recent years, some coronaviruses belonging to the *Merbecovirus* subgenus have been identified in bats [11–16], pangolins [17,18], hedgehogs [19], and minks [20], collectively termed MERS-like coronaviruses (MERS-like CoVs). Compared to MERS-CoV, which circulates in camels, MERS-like CoVs exhibit expanded host ranges, bridging zoonotic transmission risks closer to human populations. The earliest discovered *Merbecovirus*, BatCoV-HKU4 was isolated from lesser bamboo bats (*Tylonycteris pachypus*) [14], possessing the capability to utilize human DPP4 as a receptor. Specifically, the SM3A strain of BatCoV-HKU4 has demonstrated the capacity to infect hDPP4-transgenic mice [12]. However, these viruses generally exhibit weak receptor-binding affinity or occasionally necessitate exogenous trypsin treatment for efficient entry into human cells [21,22]. Recently, novel HKU4-related coronaviruses (HKU4r-CoVs) including MjHKU4r-CoV [17] and Pangolin-CoV-HKU4-P251T [18] were identified in Malayan pangolins. These viruses exhibit enhanced hDPP4 affinity compared to HKU4, suggesting pangolins as potential intermediate hosts during adaption. These observations highlight the necessity for further adaptive mutations to facilitate optimal host adaptation and replication within human systems.

In this study, we focus on two MERS-like coronaviruses from the bat, BatCoV/Ii/GD/2014-422 (2014-422) and BtTp-BetaCoV/GX2012 (GX2012). GX2012, isolated in *Tylonycteris pachypus* [23], shares the same origin with HKU4. As a newly identified HKU4r-CoV, it remains unclear whether GX2012 can utilize hDPP4 as a receptor or possesses the potential for cross-species transmission. Notably, the identification of 2014-422 in the great evening bat (*Ia io*), exhibits a high genomic similarity to MERS-CoV and, through recombination analysis, appears to have acquired its spike genes from HKU4. Importantly, protein-protein interaction assays confirmed its ability to bind hDPP4, supporting the hypothesis of potential human transmission [13].

Here, we resolved the cryo-electron microscopy (EM) structures of the spike proteins of 2014-422 and GX2012 at resolutions of 2.6 Å and 3.0 Å, respectively. Comparative structural analysis revealed that the spike trimers in 2014-422 and GX2012 adopted a more compact “closed” conformation compared to MERS-CoV. We demonstrated that both viruses RBD can bind to human DPP4 through binding experiments. However, only GX2012 exhibited efficient cell entry via hDPP4 in Huh-7 cells. To elucidate the structural basis for these observations, we further determined the cryo-EM structures of the RBD-hDPP4 complexes for both viruses, revealing conserved binding modes with MERS-CoV. Detailed structural comparisons identified the interaction interface characteristics are key determinants of hDPP4 recognition and binding affinity. These findings provide a structural rationale for differential receptor usage and highlight evolutionary constraints on viral adaptation.

## Results

### DPP4 binding and pseudovirus entry efficiency

**Fig 1:**
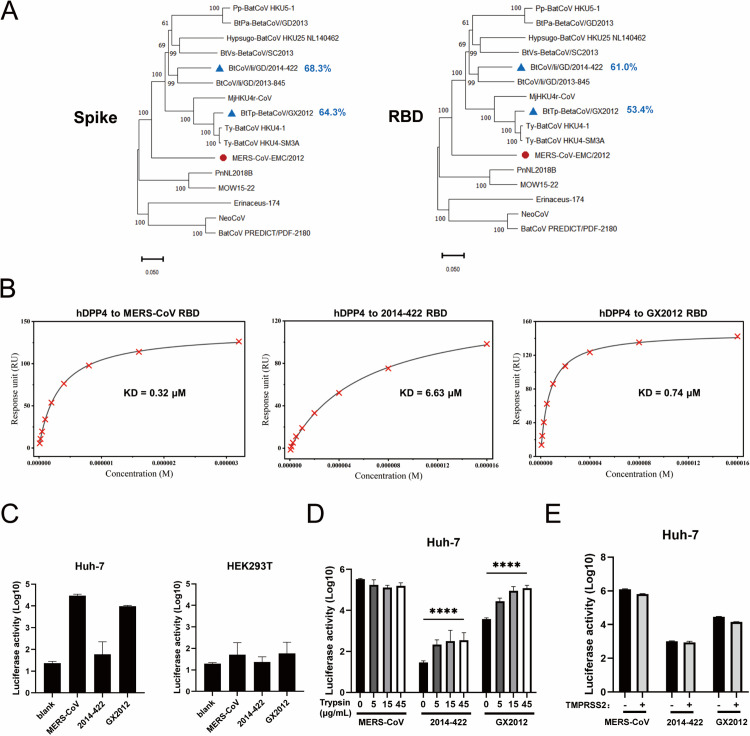
hDPP4-binding property and pseudovirus entry efficiency. (A) Phylogenetic trees derived from the amino acid sequences of merbecoviruses for Spike and RBD. Viruses characterized in this study are shown with a blue triangle in front of the taxon names and with sequence similarities backward. (B) Binding curves of immobilized hDPP4 with MERS-CoV, 2014-422, and GX2012 RBDs. Data are shown as red crosses, and the best fit of the data to a 1:1 binding model is shown as black lines. (C) The cell entry efficiency of pseudoviruses as measured by luciferase activity. Equivalent amounts of MERS-CoV, 2014-422, and GX2012 pseudoviruses were used to infect Huh-7 cells. 293T cells were used as mock. Data are presented as mean values ± SD of five replicates. (D) MERS-CoV, 2014-422, and GX2012 pseudoviruses were treated with indicated concentrations of trypsin (5, 15, and 45 µg/mL) prior to infection of Huh-7 cells. Data are presented as mean values ± SD of five replicates. ****P < 0.0001 (two-tailed unpaired t-test). (E) Pseudovirus entry assays of MERS-CoV, 2014-422, and GX2012 were performed in WT Huh-7 cells or Huh-7 cells overexpressing TMPRSS2. Data are presented as mean values ± SD of five replicates.

To elucidate the evolutionary relationships among merbecoviruses, we conducted a phylogenetic analysis using representative human and animal coronavirus sequences. Maximum-likelihood phylogenetic trees based on spike and RBD sequences revealed that both 2014-422 and GX2012 are closely related to MERS-CoV (Fig 1A). Sequence similarities among these merbecoviruses were generally low, with spike sequences showing 50%–68% identity and RBD sequences ranging from 32% to 61%. Notably, 2014-422 is the currently identified coronavirus with the highest sequence homology to MERS-CoV in both spike (68.3%) and RBD (61.0%) sequences.

In contrast, GX2012 demonstrates a closer relationship to HKU4, with spike and RBD sequence similarities of 95.7% and 91%, respectively. GX2012, along with previously identified HKU4-SM3A and MjHKU4r-CoV, belongs to the HKU4r-CoV clade and exhibits a higher sequence homology to HKU4 than MjHKU4r-CoV.

Surface plasmon resonance (SPR) experiments were conducted to assess the binding of the RBDs of MERS-CoV, 2014-422, and GX2012 to hDPP4 (Fig 1B). Since the SPR curves showed fast-on/fast-off kinetics, the binding affinity was determined using steady-state equilibrium analysis (S1 Fig). The results indicated that the MERS-CoV RBD bound to hDPP4 with an affinity of 0.32 μM, whereas the RBDs of 2014-422 and GX2012 demonstrated lower binding affinities. Specifically, the RBD of 2014-422 exhibited an affinity of 6.63 μM for hDPP4, representing a 21-fold reduction compared to MERS-CoV, while GX2012 showed an affinity of 0.74 μM, indicating a 2-fold reduction.

Cell entry efficiency is a key determinant of viral infectivity. To evaluate the cell entry efficiency of these MERS-like coronaviruses, we employed an HIV-based pseudovirus system expressing the spike proteins of MERS-CoV, 2014-422, and GX2012 (Fig 1C). Huh-7 cells, known for their high endogenous expression of hDPP4, were used as the target cell line [24]. Besides, HEK293T cells lack detectable endogenous expression of hDPP4 [25], and are therefore used as a negative control. Equal amounts of pseudotyped viruses were used to infect Huh-7 cells, with successful incorporation of spike proteins into pseudoparticles confirmed by Western blotting (S2A Fig). The spike glycoproteins of 2014-422, GX2012, and MERS-CoV were efficiently incorporated into pseudoparticles. MERS-CoV exhibits a distinct S1/S2 cleavage band, while GX2012 displays a faint cleavage band, and no cleavage band was observed in 2014-422. The results demonstrated that, in addition to MERS-CoV pseudoviruses, which efficiently utilized hDPP4 to infect Huh-7 cells, GX2012 pseudoviruses exhibited similar entry efficiency. In contrast, pseudoviruses bearing the 2014-422 spike protein showed minimal detectable entry into Huh-7 cells, indicating a significant reduction in cell entry capability.

The presence of host proteases can modulate spike cleavage processing and thereby influence viral entry. In previous studies of HKU4, exogenous trypsin enables efficient entry into human cells [22]. Accordingly, we treated MERS-CoV, 2014-422, and GX2012 pseudoviruses with graded concentrations of trypsin (5, 15, and 45 µg/mL) prior to infection of Huh-7 cells (Fig 1D). Trypsin treatment had minimal impact on MERS-CoV entry, but affected the entry of both 2014-422 and GX2012. Notably, GX2012 entry was significantly increased to a level comparable to MERS-CoV upon treatment with 15 or 45 µg/mL trypsin, whereas 2014-422 showed only a ~ 1.5-fold increase and remained inefficient in entry. Western blot analysis revealed that, upon trypsin treatment, both MERS-CoV, 2014-422 and GX2012 spikes displayed enhanced S1/S2 cleavage bands (S2B Fig). In addition, we overexpressed TMPRSS2 in Huh-7 cells; western blot confirmed robust TMPRSS2 expression in Huh-7-TMPRSS2 cells and low endogenous levels in WT Huh-7 cells (S2C Fig). Pseudovirus assays demonstrated that TMPRSS2 expression did not alter the entry efficiency of MERS-CoV, 2014-422, or GX2012 (Fig 1E).

### Overall structures of 2014-422 and GX2012 S glycoproteins

The extracellular domain proteins of the 2014-422 and GX2012 spike proteins were expressed using the Bac-to-Bac baculovirus system in Hi5 insect cells and purified by size exclusion chromatography. SDS-PAGE analysis revealed that the monomeric sizes of both spike proteins were approximately 150 kDa (S3 Fig). We determined the trimeric structures of the 2014-422 S and GX2012 S proteins, both of which adopted a closed conformation, with resolutions of 2.6 Å and 3.0 Å, respectively (S4 and S5 Figs and S1 Table). Unlike the previously resolved MERS-CoV S trimer structure [26,27], no open conformation was observed in these structures neither in 2D nor 3D classification.

**Fig 2:**
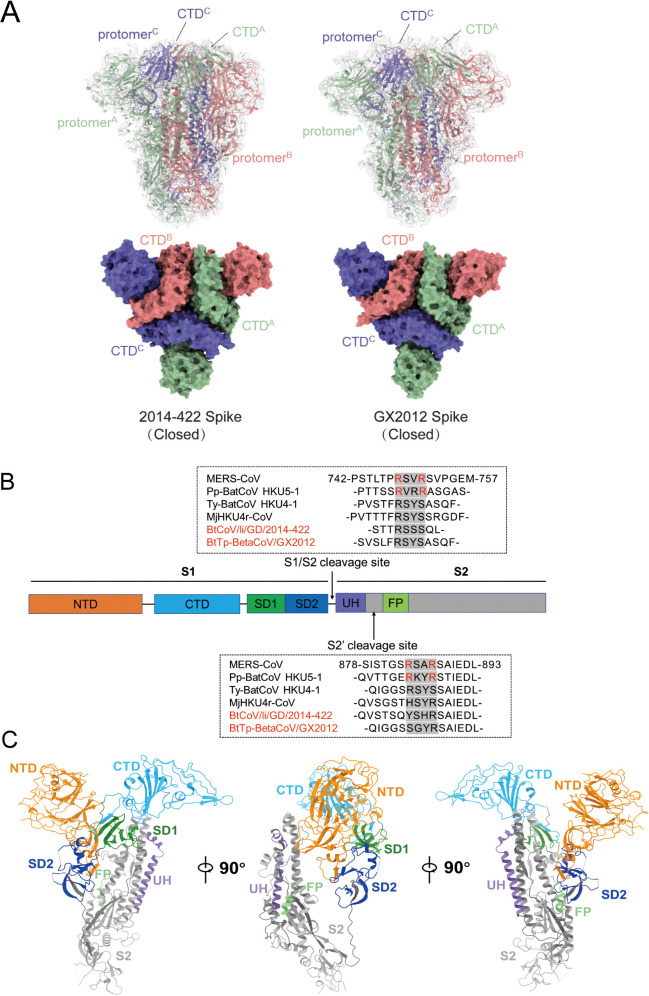
Overall structures of the 2014-422 and GX2012 S trimers. (A) (Top) Overall structures of 2014-422 and GX2012 S trimers are shown in side views, with cryo-EM maps presented as semitransparent surfaces. (Bottom) Top views of 2014-422 and GX2012 S trimers are shown in surface. Three protomers are colored coral, green, and slate blue, respectively. The locations of CTDs are indicated. (B) Segmentation of 2014-422 and GX2012 S protomer. Structural segments are shown as boxes with the width related to the length of the amino acid sequence. The position of the S1/S2 and S2′ cleavage sites are indicated by arrows. (C) Overall structure of the 2014-422 S protomer. As 2014-422 and GX2012 spikes have similar overall structures, only the 2014-422 protomer was thus used to show detailed structures.

The overall architecture and domain composition of the 2014-422 and GX2012 S trimer are consistent with those of other pre-fusion coronaviral S proteins, adopting a mushroom-like shape (Fig 2A). The three protomers of the trimer position their RBDs toward the N-terminal domain (NTD) and RBD of adjacent protomers in a counterclockwise orientation. Each protomer consists of two subunits: the receptor-recognizing S1 subunit and the membrane-fusing S2 subunit. The S1 subunit includes the NTD, CTD (RBD), and two subdomains, SD1 and SD2. The S1/S2 cleavage site is located between the S1 and S2 subunits, while the S2’ cleavage site is found between the upstream helix (UH) and the fusion peptide (FP) in the S2 subunit (Fig 2C). Both cleavage sites are recognized and cleaved by host protease, a crucial step in viral entry for MERS-CoV [28] and SARS-CoV-2 [29]. Sequence comparison at the two cleavage sites revealed that only MERS-CoV and HKU5 among the MERS-like coronaviruses harbor the furin recognition motif “R-X-X-R” at these positions. In contrast, several MERS-like coronaviruses, including 2014-422 and GX2012, lack the furin cleavage sites.

### 2014-422 and GX2012 S trimers adopt a compact conformation

We conducted a detailed structural comparison between the 2014-422, GX2012, and MERS-CoV spike proteins. Since only the closed conformation structures of 2014-422 and GX2012 were resolved, we selected the similarly closed MERS-CoV spike structure (PDB: 8IFN) for comparative analysis.

**Fig 3:**
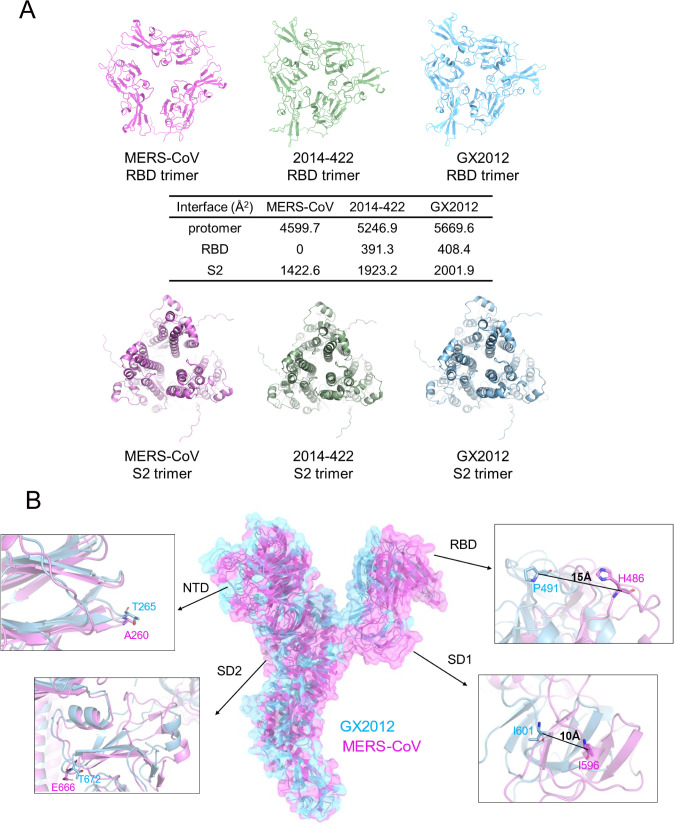
Structural comparisons between 2014-422, GX2012, and MERS-CoV S trimers. (A) The RBD and S2 trimers of closed state of 2014-422, GX2012, and MERS-CoV are shown in the top view. The buried interface calculated by Protein Interfaces, Surfaces and Assemblies (PISA) is listed in the table. (B) GX2012 and MERS-CoV S protomers are aligned by the S2 subunit. The GX2012 S protomer is in blue, and the MERS-CoV S protomer is in magenta. The measurement of structural movements in NTD, RBD, SD1 and SD2 subdomain are shown in magnify details. PDB code: MERS-CoV S, 8IFN.

Viewed from the top, the S trimers of 2014-422 and GX2012 were more compact than MERS-CoV, with significant differences in the interaction areas between the three RBDs (Fig 3A). Specifically, the buried interface area of the RBDs in 2014-422 and GX2012 is approximately 400 Å², whereas the loosely closed state of MERS-CoV exhibits minimal inter-RBD contacts. Additionally, the buried interface area between the S2 subunits of 2014-422 and GX2012 increased by approximately 500 Å² compared to MERS-CoV. Upon alignment of one S2 subunit, the other two S2 subunits in 2014-422 and GX2012 displayed substantial displacement, shifting around 10 Å toward the C3 symmetry axis. This shift resulted in a significant increase in the buried interface area between the S2 subunits, making the S1 and S2 subunits more tightly packed from top to bottom.

Further alignment of the S protomers based on S2 subunit superposition revealed that the RBD and SD1 regions in 2014–422 and GX2012 shifted significantly compared to MERS-CoV (Fig 3B). In GX2012, the RBD moved approximately 15 Å closer to the trimer axis, while the SD1 region shifted by about 10 Å. In addition, the NTD and SD2 regions were slightly displaced compared to MERS-CoV. This looser structural arrangement facilitates the transition of the MERS-CoV RBD from the down to up position more easily.

Comprehensive structural analysis revealed that the NTD, RBD, SD1, and S2 regions of the S protomer exhibit high similarity among 2014-422, GX2012, and MERS-CoV, with a root mean square deviation (RMSD) of less than or approximately equal to 1 Å (S6 Fig). However, the SD2 region shows a higher RMSD value, potentially due to differences in cleavage site presence and edge resolution limitations. Additionally, the overall compactness of the trimer structure contributes to the notable differences observed among the three viruses.

### The complex structure of the 2014-422 and GX2012 RBD with hDPP4

The results above indicated that both 2014-422 and GX2012 may utilize hDPP4 as their receptor. To gain insights into the details of their interaction, we attempted to resolve the structure of the spike protein in complex with hDPP4. The spike proteins of 2014-422 and GX2012 were mixed with hDPP4 at a 1:3 molar ratio and observed the protein particles under negative-stain EM. However, no hDPP4-bound particles were observed in the two-dimensional (2D) classification (S7 Fig). Combined with structural analysis of the spikes alone revealed a tightly closed conformation, leading to the hypothesis that the receptor hDPP4 alone may not be sufficient to induce the conformational transition of the spike from the closed to the open state. Recent studies have shown that HKU1 spikes can undergo glycan-triggered RBD opening, with 9-O-acetylated sialic acid binding to the NTD facilitating the transition from closed to open states. Notably, HKU1C spikes display a high intrinsic level of conformational plasticity, with nearly half of the RBDs adopting an open conformation in the apo state [30]. In contrast, all available MERS-like spike structures remain tightly closed.

The RBD of the spike protein is responsible for binding to the receptor hDPP4. To further investigate this interaction, we mixed the RBD proteins of 2014-422 and GX2012 with hDPP4 and resolved the RBD-hDPP4 complex structures using cryo-EM, achieving resolutions of 3.0 Å and 2.4 Å, respectively (S8 and S9 Figs and S1 Table). Since hDPP4 exists as a dimer in its natural state, we obtained the structure of one hDPP4 monomer bound to the RBD through 3D classification, though the second RBD could not be reconstructed due to resolution limitations (S10 Fig).

**Fig 4:**
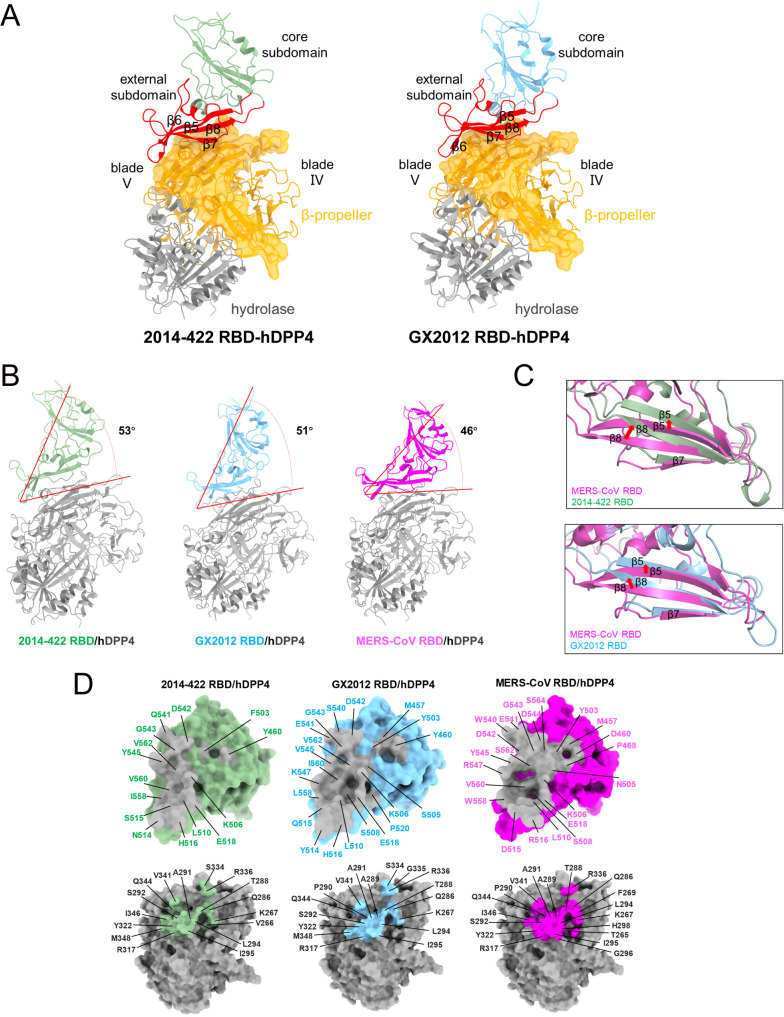
Structures of the 2014-422 and GX2012 RBD-hDPP4 complex. (A) The core subdomains of 2014-422 RBD and GX2012 RBD are individually labeled and highlighted in green and blue. The external subdomains of the RBDs are in red and the β-strands are specified by ESPript. The β-propeller and hydrolase domains of DPP4 are in orange and dark grey. The blade IV and blade V of β-propeller that engage RBD are shown in semitransparent surfaces. (B) The binding angles of 2014-422 RBD, GX2012 RBD and MERS RBD with hDPP4. Three RBDs are colored green, blue and magenta, respectively. (C) Structural comparison of RBD displacement in the RBD-hDPP4 complex. β5, β7 and β8 strands are labeled. (D) The binding interface of 2014-422 RBD-hDPP4, GX2012 RBD-hDPP4, and MERS-CoV RBD-hDPP4 (PDB:4KR0). Three RBDs and hDPP4 are shown as surfaces. The AA sequences of 2014-422 and MERS-CoV RBD are renumbered based on the GX2012 RBD for convenient structural comparison.

As observed in previous reports [31], hDPP4 monomer consists of two domains: an N-terminal eight-bladed β-propeller with an open Velcro topology and a C-terminal α/β-hydrolase domain. Similar to the previously resolved MERS-CoV RBD-hDPP4 [32,33], HKU4 RBD-hDPP4 [22], and MjHKU4r-CoV RBD-hDPP4 [34,35] complexes, hDPP4 also interacts with the 2014-422 RBD and GX2012 RBD through blade IV and blade V of the β-propeller domain (Fig 4A). The RBD is composed of two subdomains: the core subdomain, which is oriented away from hDPP4 and does not interact with it, and the external subdomain, also known as the receptor-binding motif (RBM). The RBM engages hDPP4 predominantly via the β5-β8 strands and the β5-β6 loop.

While the overall binding modes of the RBDs to hDPP4 are similar, detailed comparisons reveal some differences. In terms of binding angles, the 2014-422 RBD and GX2012 RBD bind to hDPP4 at angles of 53° and 51°, respectively, whereas the MERS-CoV RBD binds at a smaller angle of approximately 46° (Fig 4B). Alignment of hDPP4 in the complex structures revealed significant deviations in the β-strands, particularly β5, β7, and β8, of 2014-422 and GX2012 RBM, which shifted away from the hDPP4 binding interface. Notably, the β8 strand showed the largest deviation, with an upward rotation of approximately 11° in 2014-422 and 8° in GX2012 (Fig 4C). These differences directly result in varying interaction surfaces between the RBDs and hDPP4. The MERS-CoV RBD, with the smallest binding angle, is positioned closest to hDPP4, interacting through 22 amino acids on the RBD and 19 on hDPP4, resulting in the largest binding surface area (BSA) of 1963 Å² (926 Å² on RBD and 1037 Å² on hDPP4). In contrast, the 2014-422 RBD, with the largest binding angle, is positioned farther from hDPP4, interacting through 15 amino acids on the RBD and 16 on DPP4, yielding in the smallest BSA of 1495 Å² (715 Å² on 2014-422 RBD and 780 Å² on hDPP4), a 24% reduction compared to MERS-CoV. The GX2012 RBD, with an intermediate binding angle, also exhibits an intermediate interaction area (BSA = 1690 Å²), a reduction of 14% compared to MERS-CoV, through interactions involving 21 amino acids on the RBD and 17 on hDPP4 (Fig 4D and S2 and S3 Tables). Residues located at the RBD-hDPP4 interface were identified using a 4.0 Å distance cutoff.

### Atomic details comparation at the hDPP4 binding interface between 2014-422, GX2012 and MERS-CoV RBD

**Fig 5:**
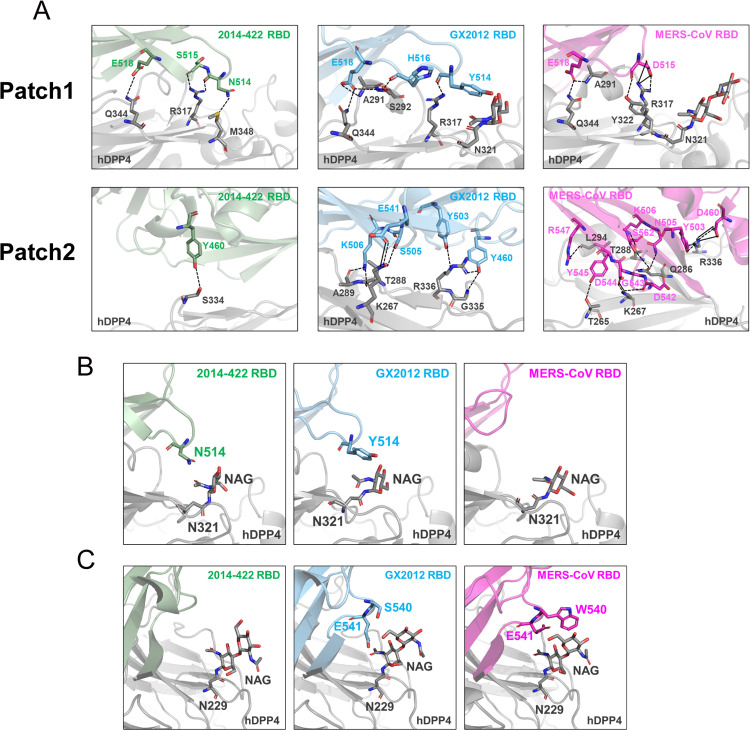
Structural details of the 2014-422 RBD-hDPP4, GX2012 RBD-hDPP4, and MERS-CoV RBD-hDPP4 complexes. (A) The three complexes are structurally aligned, and their interaction networks are divided into Patch1 and Patch2 for comparison analyses. The AA sequences of the 2014-422 and MERS-CoV RBDs were renumbered for convenient structural comparison based on the GX2012 RBD. hDPP4 is colored in dark grey, and the 2014-422, GX2012, and MERS-CoV RBDs are colored in green, blue, and magenta, respectively. The black dashes present H-bonds and the black solid lines present salt bridge. (B) The RBDs interactions with N321 glycan of hDPP4. (C) The RBDs Interactions with N229 glycan of hDPP4.

Based on the analysis of the overall structure of RBD-hDPP4, we further explored the interaction details between the three RBDs and hDPP4. Hydrogen bonds and salt bridges play a critical role in the stability of protein-protein interactions. We divided the interaction interface between RBD and hDPP4 into two regions, Patch1 and Patch2, for analysis. Patch1 is located in the β5-β6 loop region of RBM, while Patch2 is situated in the β5-β8 strands region of RBM (Fig 5A).

In the 2014-422 RBD-hDPP4 complex, a total of 5 hydrogen bond interactions were formed. Specifically, in Patch1, 2014-422 RBD interacts with hDPP4 through S515 and E518, forming one hydrogen bond each with R317 and Q344 of hDPP4. Additionally, N514 forms one hydrogen bond each with M348 and R317 of hDPP4. In Patch2, Y460 forms a hydrogen bond with S334 of hDPP4. In the GX2012 RBD-hDPP4 complex, a total of 12 hydrogen bonds and 1 salt bridge interaction were formed. In Patch1, GX2012 RBD interacts with hDPP4 through Y514 and H516, forming one hydrogen bond each with R317 and S292 of hDPP4. E518 forms hydrogen bonds with A291, S292, and Q344 of hDPP4. In Patch2, Y503 and S505 form hydrogen bonds with T288 and R336 of hDPP4, respectively. Y460 interacts with G335 and R336 of hDPP4, forming hydrogen bonds. K506 forms hydrogen bonds with T288 and A289 of hDPP4. E541 forms one hydrogen bond and one salt bridge interaction with K267 of hDPP4. In the MERS-CoV RBD-hDPP4 complex, a total of 14 hydrogen bonds and 6 salt bridge interactions were formed. In Patch1, MERS-CoV RBD interacts with hDPP4 through E518, forming two hydrogen bonds with A291 and Q344 of hDPP4. D515 forms one hydrogen bond and two salt bridge interactions with R317 of hDPP4, and also forms one hydrogen bond with Y322. Patch2 shows extensive interactions with D460, Y503, N505, K506, D542, G543, D544, Y545, R547, and S562 of MERS-CoV RBD interact with multiple amino acids of hDPP4, forming hydrogen bonds and salt bridge interactions. Specifically, D460 forms one hydrogen bond and two salt bridges with R336 of hDPP4. D544 forms one hydrogen bond and one salt bridge interaction with K267 of hDPP4.

Extensive electrostatic interactions and hydrogen bonding networks contribute to the stability of the interaction between the RBD and hDPP4. Hydrophobic interactions further contribute to the stability of the protein-protein interface. In the study of the MERS-CoV RBD-hDPP4 interaction, a shallow concave region on the outer surface, referred to as Patch2, was identified [33]. This region is formed by the β6 strand, C-terminal ends of β5 and β7, N-terminal end of β8, and the β5-β6 connecting loop. This concave area accommodates a short α-helix located between blade IV and blade V of hDPP4, forming a hydrophobic core (S11 Fig). The hydrophobic core includes residues L510, W558 and V560 from RBD, and residues L294, and I295 from hDPP4. This hydrophobic core is surrounded by hydrophilic amino acids. Importantly, this hydrophobic core is preserved in the interactions of the 2014-422 RBD-hDPP4 and GX2012 RBD-hDPP4 complexes, with all three RBDs exhibiting similar hydrophobic grooves in Patch2 that serve to stabilize the interaction.

Additionally, we observed two glycan sites, N321 and N229, from hDPP4 at the interaction interface (Fig 5B and 5C). Sequence alignment revealed that 2014-422 and GX2012 possess an extra amino acid at position 514 compared to MERS-CoV, with Asparagine and Tyrosine, respectively. This sequence divergence results in an extended β5-β6 loop in 2014-422 and GX2012, enabling the amino acid at position 514 to interact with the N321 glycan. This interaction may pull the RBD of 2014-422 and GX2012 toward the N321 glycan, inducing structural displacement (Fig 5B). Near the N229 glycan of hDPP4, W540 and E541 of MERS-CoV, as well as S540 and E541 of GX2012, can interact with the N229 glycan, while 2014-422 is too distant to engage with this glycan. This differential interaction stabilizes GX2012 RBD binding to hDPP4, resulting in a smaller RBD deviation compared to 2014-422 (Fig 5C).

We generated deletion of residue 514 mutant in 2014-422 RBD (2014-422-d514 RBD). The 2014-422-d514 RBD yielded a high-purity protein and exhibited approximately a 6.5-fold increase in binding affinity to hDPP4 compared with the wild-type (WT) RBD (S12A and S12B Fig). We used Alphafold3 [36] to predict the complex structure of the 2014-422-d514 RBD-hDPP4 with a high-confidence (S13A Fig). Structural alignment based on hDPP4 revealed that the 2014-422-d514 RBD adopts a noticeable shift in the binding orientation toward the receptor compared with the WT 2014-422 RBD (S13B Fig). This repositioning increases interfacial contacts, including additional hydrogen bonds and salt bridges, and expands the buried surface area, providing a structural basis for the enhanced binding affinity (S13C Fig). In pseudovirus entry assays using Huh-7 cells, we introduced reciprocal mutations: insertion of residue 514 into the MERS-CoV spike (MERS-CoV-N514) and deletion of residue 514 in the 2014-422 spike (2014-422-d514). These mutations had no detectable effect on MERS-CoV and 2014-422 pseudovirus entry (S12C Fig).

## Discussion

Coronaviruses, particularly SARS-CoV, MERS-CoV, and SARS-CoV-2, have posed significant threats to public health in recent decades due to their widespread transmission. Bats are the main natural reservoirs for coronaviruses [11,37]. Despite limited direct human-bat interactions, cross-species transmission from bats to intermediate hosts, and subsequently to humans, remains a critical concern. Recently, two distinct coronavirus clades, represented by HKU4 and HKU5, have been discovered within the *Merbecovirus* subgenus, utilizing DPP4 and ACE2 as their respective receptors [15,22,38].

In this study, we focused on two Merbecoviruses, 2014-422 and GX2012, isolated from great evening bats (*I. io*) and lesser bamboo bats (*Tylonycteris pachypus*), respectively. Sequence analysis of spike and RBD revealed that 2014-422 exhibits the highest similarity to MERS-CoV among coronaviruses identified to date, with sequence identities of 68.3% for the spike protein and 61.0% for the RBD. GX2012, a member of HKU4-related coronaviruses, shares sequence similarities of 64.3% and 53.4% with MERS-CoV spike and RBD, respectively. We determined that both 2014-422 and GX2012 RBDs bind to hDPP4 with weaker affinity compared to MERS-CoV RBD. Pseudovirus entry assays showed that only GX2012 efficiently utilized hDPP4 to infect Huh-7 cells. Exogenous trypsin treatment efficiently promoted S1/S2 cleavage of both MERS-CoV, 2014-422 and GX2012 spikes. Under higher trypsin concentrations, GX2012 reached entry efficiencies comparable to those of MERS-CoV, whereas 2014-422 remained unable to mediate efficient entry. This disparity indicates that, beyond proteolytic priming, additional restrictions likely impede the productive entry of 2014-422 into human cells. Moreover, overexpression of TMPRSS2 failed to enhance entry of any of the three spikes, suggesting that TMPRSS2 does not efficiently engage these spikes under our experimental conditions. Previous studies indicated that HKU4 requires trypsin treatment to infect human cells [21,22], suggesting that GX2012, despite sharing the same bat origin, has evolved further compared to HKU4. In pangolin HKU4r-CoVs, including MjHKU4r-CoV and pangolin-CoV-HKU4-P251T, utilize hDPP4 to infect human cells with higher affinity [17,18]. This finding provides evidence for the evolutionary role of pangolins as intermediate hosts in HKU4r-CoVs transmission. Overall, HKU4r-CoVs appear to acquire the ability to infect human cells through two evolutionary pathways: within bats and via pangolins as intermediate hosts. Although 2014-422 RBD can bind to hDPP4, its failure to enter cells in pseudovirus assays likely reflects the influence of additional complex factors, such as the dominant conformation of spike protein and proteolytic cleavage. Despite exhibiting higher homology in both the spike and RBD compared to GX2012 and other HKU4r-CoVs, the marginal improvement observed in 2014-422 does not confer an advancement in evolutionary proximity to MERS-CoV.

The spike protein on the surface of coronaviruses plays a critical role in receptor recognition and membrane fusion, facilitating cellular entry. In several coronaviruses, the transition of the RBD from down to up position has been identified as a key step in receptor recognition. Using cryo-EM, we observed that the spike proteins of 2014-422 and GX2012 exhibited closed state, with all three RBDs in the down conformation. While the overall structure and components resemble the previously characterized MERS-CoV spike, the latter was predominantly observed in the open state [26]. Previously, another group reported the cryo-EM structure of 2014-422 spike bound to the antibody JC57-11 [39]. The antibody binds to the closed conformation of 2014-422 spike, and aligning our 2014-422 spike trimer with the structure (PDB: 8SAK) revealed a high degree of similarity, with the trimer fitting well together and maintaining a consistent level of compactness (S14 Fig). This indicates that the closed conformation of 2014-422 spike is relatively stable, and no open conformation has been observed in vitro to date.

Comprehensive structural comparisons were conducted with MERS-CoV spike. We observed that the closed states of 2014-422 and GX2012 spike trimers adopted a compact conformation for both S1 and S2 subunits, in contrast to the loosely closed conformation observed in the MERS-CoV spike trimer. Furthermore, no binding of 2014-422 and GX2012 spike proteins to hDPP4 was detected in negative-staining EM, suggesting that the compact conformation of these spikes restricts RBD upturning, thereby hindering interaction with the hDPP4 receptor. Additionally, the MERS-CoV spike contains S1/S2 and S2’ furin cleavage sites, which are crucial for viral entry and have been shown to result in S1 and S2 cleavage bands in Western blot analysis [17]. These furin cleavage sites have been shown to play a crucial role in the viral entry process of MERS-CoV and SARS-CoV-2, potentially being associated with spike conformational changes [28,29]. It has been observed that neither 2014-422 nor GX2012 possess canonical S1/S2 and S2’ furin cleavage sites. However, a faint cleavage band was detected in GX2012 via Western blot analysis (S2A Fig), suggesting the potential presence of a non-canonical cleavage site at an alternative location. The presence of potential proteolytic cleavage sites may facilitate the efficient entry of GX2012 pseudovirus into Huh-7 cells. In the study of MjHKU4r, a potential furin cleavage site (RQQR) in SD1 was identified, and its mutation resulted in the disappearance of the cleavage band [17]. In GX2012 and 2014-422, the corresponding sequence is RNQR and KQQR, respectively. However, the site function remains to be determined. In SARS-CoV-2, the D614G mutation in SD2 enhances infectivity by increasing the proportion of spike in the open conformation [40]. In our previous work, the Y623H mutation in SD2 was shown to induce RBD upturning, facilitating the open conformation in RsSHC014 and enabling infection of 293T-ACE2 cells [41]. This suggests that SD2 may regulate RBD conformation dynamics and further modulate spike conformational states. The SD2 role in MERS-like coronaviruses remains to be investigated.

We further analyzed the cryo-EM structures of the RBD of 2014-422 and GX2012 in complex with hDPP4. The binding modes of the two RBD-hDPP4 complexes are similar to those of previously reported structures, including MERS-CoV RBD-hDPP4 [32,33], HKU4 RBD-hDPP4 [22], and MjHKU4r-CoV RBD-hDPP4 [34,35], primarily involving interactions between the β5-β8 strands and the β5-β6 loop of the RBD and blades IV and V of the hDPP4 β-propeller domain (Fig 4A). Detailed comparisons with the MERS-CoV RBD-hDPP4 complex revealed that variations in the binding angles between the RBDs and hDPP4 influence the BSA, which consequently affect the number of amino acid contacts and non-covalent bond formations, ultimately influencing the binding affinity between the RBD and hDPP4. The MERS-CoV RBD-hDPP4 complex, with the largest BSA, forms the most hydrogen bonds and salt bridges, stabilizing the interaction and yielding the strongest binding affinity (Fig 1B and S2 Table). In contrast, the reduced BSA of the 2014-422 RBD results in weaker binding affinity (Fig 1B).

Our structural analysis indicates that the distinct RBD binding angles of 2014-422 and GX2012 may arise from an additional amino acid at position 514 in their β5-β6 loop, compared to MERS-CoV. This extra residue facilitates interaction with the N321 glycan on hDPP4, pulling the RBD of both 2014-422 and GX2012 toward N321, leading to a structural shift away from the hDPP4 binding interface. The primary structural adjustments occur in the β5, β7, and β8 strands of the RBM. Notably, GX2012 RBD maintains interaction with the N229 glycan on hDPP4, similar with MERS-CoV, reducing RBD tilting and enhancing binding stability. Structure-guided mutagenesis showed that deletion of residue 514 markedly enhanced the binding affinity of the 2014-422 RBD for hDPP4. Consistent with these findings, AlphaFold3 modeling confirmed enhanced hDPP4 binding to 2014-422 d514 RBD. However, this increased affinity did not translate into improved pseudovirus entry, underscoring the complexity of cell-based entry mechanisms.

Notably, during the preparation of this manuscript, an article describing the cryo-EM structures of the 2014-422 RBD-hDPP4 and MjHKU4r RBD-hDPP4 was published online [42]. Consistent with their findings, 2014-422 was also reported to bind hDPP4 with lower affinity, and mutational analyses identified several key residues at the interaction interface. RBM swap experiments further suggested limited plasticity of *Merbecovirus* RBM regions. In contrast, through structural comparison with the MERS-CoV RBD-hDPP4 complex, our analysis identified residue 514 as an important site influencing hDPP4 binding to 2014-422 RBD. We also compared the structures of HKU4, MjHKU4r, and GX2012 RBD-hDPP4 complexes. Phylogenetic analysis grouped MjHKU4r and GX2012 as HKU4-related coronaviruses (Fig 1A). Previous SPR assays showed that the binding affinity of MjHKU4r RBD to hDPP4 was approximately 20-fold greater than that of HKU4. Pseudovirus infection assays further demonstrated that MjHKU4r pseudoviruses exhibited significantly higher infection efficiency than HKU4 [35]. Despite similar BSAs (~1660 Å²) for all three complexes, the number of interacting amino acids, hydrogen bonds, and salt bridges were comparable (S15A Fig). However, the binding epitopes differed substantially. Among the 19 interacting residues in HKU4 RBD-hDPP4, 6 were located in the core subdomain, while the remaining 13 were in the RBM region. In contrast, 17 of the 19 interacting residues in MjHKU4r and 19 of the 21 in GX2012 were located in the RBM region, with a higher proportion of RBM residues contributing to the interaction. The residues in the core region were more conserved (S15B Fig). This discrepancy may explain why HKU4 RBD-hDPP4, despite a larger interaction area, exhibits lower binding affinity and infection efficiency compared to MjHKU4r. This analysis further supports the evolutionary trajectory of HKU4-related coronaviruses.

## Materials and methods

### Protein expression and purification

The cDNAs encoding MERS-CoV spike (GenBank: JX869059.2), 2014-422 spike (GenBank: MG021452), GX2012 spike (GenBank: KJ473822.1) and the mutant spikes were synthesized by GenScript and were cloned into pcDNA3.1 vectors (Invitrogen) with a C-terminal Strep-tag. The spike ectodomains of 2014-422 (1–1288), and GX2012 (1–1291) were cloned into the pFastBac-Dual vector. All of these spike constructs featured a C-terminal foldon-tag for trimerization and a Twin-Strep-tag for purification for protein stabilization. The RBDs of MERS-CoV (367–589), 2014-422 (371-593), GX2012 (372-594), the mutant RBDs and the extracellular domains (39–766) of hDPP4 were separately inserted into the pCAG vector, with an N-terminal CD4 signal peptide for secretion and a C-terminal 6 × his tag for purification.

The spike ectodomains were produced in Hi5 insect cells using the Bac-to-Bac baculovirus system (Invitrogen). Briefly, the amplified high-titer baculoviruses were used to infect Hi5 cells at a density of 2 × 10^6^ cells/mL. After 60 h, the supernatants were harvested. The spike proteins within the supernatants were captured using StrepTactin beads (IBA) and subsequently purified by gel-filtration chromatography using a Superose 6 column (GE Healthcare) with HEPES-buffered saline (HBS) solution (10-mM HEPES, pH 7.2, 150-mM NaCl).

Both the RBDs and hDPP4 were produced in HEK293F cells. In brief, cell cultures were transfected with 1 mg of plasmids/L of culture with a density of 2 × 10^6^ cells/mL, using polyethylenimine (Sigma). After 72 h, the supernatants were harvested. The RBDs or hDPP4 within the supernatants were captured using Ni-NTA resin (GE Healthcare) and further purified by gel-filtration chromatography using a Superdex 200 column (GE Healthcare) with HBS buffer (10-mM HEPES, pH 7.2, 150-mM NaCl).

### Surface plasmon resonance experiments

The hDPP4 were immobilized on a CM5 sensor chip (GE Healthcare) at around 700 response units using Biacore S200 (GE Healthcare). The blank channel on the chip was used as the negative control. The running buffer was composed of 10-mM HEPES at pH 7.2, 150-mM NaCl, and 0.05% (vol/vol) Tween-20. Serial dilutions of MERS-CoV, 2014-422, GX2012 RBD and the mutant RBDs were sequentially flowed through the CM5 chip. The resulting data were analyzed using Biacore Evaluation Software (GE Healthcare) by fitting to a 1:1 binding model.

### Pseudovirus entry assays

The pseudoviruses were generated by co-transfecting HEK293T cells (ATCC) with pcDNA3.1 vectors encoding S glycoproteins and human immunodeficiency virus backbones expressing firefly luciferase (pNL4-3-R-E-luciferase). The “blank” sample is HEK293T cells transfected with the pcDNA3.1 empty vector instead. At 48 h post-transfection, the cell culture medium was collected for centrifugation at 2,000 rpm for 10 min, and then the supernatant was subsequently aliquoted and stored at −80°C for further use. For the trypsin assays, the pseudoviruses prepared in serum-free-media (SFM) were incubated with 3, 15, and 45 µg/mL TPCK-treated trypsin (Sigma-Aldrich) for 10 min at 37°C. Trypsin was inactivated with 100 μg/mL soybean trypsin inhibitor (STI, Sigma-Aldrich) for 5 min at room temperature. The same amount of pseudoviruses was used to infect Huh-7 cells (ATCC). HEK293T cells was also infected as negative control. The infected cells were lysed 48 h after infection, and viral entry efficiency was measured as luciferase activity by a Fire-Lucy assay kit (Vigorous Biotechnology Beijing Co.,Ltd) using Prism (v.8.3.0, GraphPad) for analysis.

### Western blotting

Whole-cell lysates of Huh-7 cells transfected with plasmids encoding TMPRSS2 protein were harvested using radioimmunoprecipitation assay (RIPA) lysis buffer (50-mM Tris-HCl, pH 8.0, 150-mM NaCl, 1% NP-40, 0.5% sodium deoxycholate, and 0.1% SDS), with the supplement of phenylmethylsulfonyl fluoride (PMSF) (Amresco) and protease inhibitor cocktail (Thermo Fisher). Pseudotyped particles were concentrated through ultracentrifugation at 30,000 rpm for 2 h over a 20% sucrose cushion, and then resuspended in the radioimmunoprecipitation assay (RIPA) lysis buffer (50-mM Tris-HCl, pH 8.0, 150-mM NaCl, 1% NP-40, 0.5% sodium deoxycholate, and 0.1% SDS). The concentrated pseudotyped particles were resolved by 8% SDS-PAGE, and transferred onto Nitrocellulose (NC) membranes. The blots were blocked with 5% non-fat milk in Tris-buffered saline with Tween 20 (TBST) at room temperature for 1 h, and then probed with primary antibodies anti-Strep (ABclonal, AE066) and anti-p24 (Abcam, ab32352) overnight at 4°C. The blots were washed three times with TBST, followed by incubation with horseradish peroxidase (HRP)-conjugated secondary antibodies for 1 h at room temperature. After washing three times with TBST, the membranes were visualized using Gelview 6000plus (Guangzhou Biolight Biotechnology Co., Ltd., China).

### Negative-staining EM

The purified 2014-422 and GX2012 spike ectodomains were mixed with hDPP4 on ice for several minutes at a molar ratio of 1:3. These S-hDPP4 mixtures (5 μL, 0.05 mg/mL) were applied onto glow-discharged grids with a continuous carbon layer (Beijing Zhongjing keyi Technology Co., Ltd.) and subsequently negatively stained using 2% uranyl acetate solution. These grids were examined under an FEI Tecnai Spirit electron microscope, equipped with an FEI Eagle 4k CCD camera. Images were manually collected at a magnification of ×42,000, with a defocus range between 1.5 and 1.8 μm, corresponding to a pixel size of 5.29 Å. For each sample, about 50 images were collected. EMAN [43] was used for image format conversion, while CyroSPARK [44] was employed for particle picking, particle extraction, and 2D classification.

### Cryo-EM sample preparation and data collection

Aliquots of spike ectodomains (4 μL, 1.3 mg/mL) were applied onto glow-discharged holey carbon grids (Quantifoil, Au 300 mesh, R1.2/1.3) and then blotted and plunge-frozen into liquid ethane using an FEI Vitrobot Mark IV.

Images were recorded using an FEI Titan Krios microscope operating at 300 kV with a Gatan K3 Summit direct electron detector (Gatan) at Tsinghua University. A total of 1,586 movies for 2014-422 S, 1,288 movies for GX2012 S, 3,965 movies for 2014-422 RBD-hDPP4, and 1,827 movies for GX2012 RBD-hDPP4 were collected, with a defocus range between 1.5 and 1.8 μm. Each movie has a total accumulated exposure of 50 e−/Å2 fractionated in 32 frames of 175-ms exposure. The final images were binned twofold to a pixel size of 1.0979 Å for 2014-422 S, GX2012 S, and 2014-422 RBD-hDPP4, and 1.0742 Å for GX2012 RBD-hDPP4. Data collection statistics are summarized in S1 Table.

### Cryo-EM data processing

Motion correction [MotionCor2 v.1.2.6] [45], CTF estimation [GCTF v.1.18] [46] and non-templated particle picking (Gautomatch v.0.56, https://github.com/JackZhang-Lab/ Gautmatch) were automatically carried out by TsingTitan.py program during the data collection process. For all proteins, sequential data processing was performed using cryoSPARC (v.3.3.2) [44]. For 2014-422 S, particles were subjected to two rounds of 2D classification, and the best selected 482,868 particles from 2D classification were applied for the initial model and 3D classification. The best class (346,468 particles) from 3D classification were subjected to 3D auto-refine with C3 symmetry to generate a density map with a resolution of 2.59 Å. For GX2012 S, particles were subjected to two rounds of 2D classification, and the best selected 146,555 particles from 2D classification were applied for the initial model and 3D classification. The best class particles (87,577 particles) were subjected to 3D auto-refine with C3 symmetry to generate a density map with a resolution of 3.02 Å. For 2014-422 RBD-hDPP4, 4,095 exposures were imported into cryoSPARC. Patch CTF estimation was used followed by blob picker. Two rounds of 2D classifications were excuted to remove low-quality particles. Ab initio reconstruction, heterogeneous refinement and 3D classifications with a focused mask were used to obtain RBD-bound hDPP4. Although we classified single-bound and double-bound hDPP4, only single-bound was focused on for later processing due to the weaker density of double-bound form. The best 2014-422 RBD-bound particles (591,107 particles) were subsequently subjected to 3D auto-refine to generate a density map with a resolution of 3.0 Å. Finally, 2014-422 RBD was subtracted and locally refined. For GX2012 RBD-hDPP4, raw movies were imported into cryoSPARC. Patch motion correction as well as patch CTF estimation was used to obtained drift-corrected exposures. Blob picker was used sequentially. After two rounds of 2D classifications, 1,814,108 particles were selected. Then, ab initio reconstruction and heterogeneous refinement were used to obtain intial volume. 3D Classification with spherical mask focused on RBD resulted in apo-hDDP4 and RBD-bound hDPP4. The best GX2012 RBD-bound particles (447,216 particles) were subsequently subjected to 3D auto-refine to generate a density map with a resolution of 2.4 Å. The reported resolutions were estimated with a gold-standard Fourier shell correlation cutoff of 0.143. Finally, GX2012 RBD was subtracted and locally refined. Data processing statistics are summarized in S1 Table.

### Model building and refinement

The initial models for all structures were generated using the CryoNet [47] and fitted into the corresponding map using UCSF Chimera (v.1.14) [48]. Manual model rebuilding was carried out using Coot (v.0.9.4) [49] and refined with PHENIX (v.1.20.1) [50] real-space refinement. The quality of the final model was analyzed with Molprobity [51] and EMRinger [52]. The validation statistics of the structural models are summarized in S1 Table. All structural figures were generated using PyMOL (v.2.5.1) [53] and ChimeraX (v.1.2.5) [54].

### AlphaFold3 structure prediction

The complex structures of the 2014-422-d514 RBD in complex with hDPP4 were predicted using AlphaFold3 (AF3) [36]. Full-length amino acid sequences of 2014-422-d514 RBD and hDPP4 ectodomain were used as inputs. For each RBD-hDPP4 pair, multiple models were generated under default AF3 settings without experimental restraints. The highest-confidence model, as indicated by predicted aligned error (PAE) values at the protein-protein interface and overall confidence scores, was selected for structural analysis. Structural alignments and interface analyses were performed using PyMOL and ChimeraX.

## Supporting information

S1 Fig SPR sensorgrams of hDPP4 with RBDs. SPR sensorgrams of immobilized hDPP4 binding to MERS-CoV (A), 2014-422 (B) and GX2012 (C) RBDs. Data are shown as colored lines.
(DOCX)

S2 Fig Western blot analyses of spike pseudovirus incorporation and TMPRSS2 expression. (A) Western blot analysis of MERS-CoV, 2014-422 and GX2012 spike pseudotype incorporation. All spike proteins tagged with C-terminal Strep-tag. The sample transfected with the pcDNA3.1 empty vector was used as a negative control. p24 used as the loading control. (B) Western blot analysis of MERS-CoV, 2014-422 and GX2012 spike with 15 µg/mL trypsin treatment pseudotype incorporation. (C) Western blot analysis of Huh-7 cells transfected with TMPRSS2 and Huh-7 WT cells. TMPRSS2 protein tagged with C-terminal Flag-tag. β-actin used as the loading control. (D) Western blot analysis of Spike mutants pseudotype incorporation. All spike proteins tagged with C-terminal Strep-tag. p24 used as the loading control.
(DOCX)

S3 Fig Expression and purification of 2014-422 and GX2012 Spike proteins. Size-exclusion chromatography profile and SDS-PAGE analysis of the gel-filtration elution. (A) 2014-422 Spike trimer. (B) GX2012 Spike trimer.
(DOCX)

S4 Fig Cryo-EM analysis of 2014-422 spike trimers. (A) A representative cryo-EM micrograph from 1,586 micrographs. (B) 2D class averages of characteristic projection views of cryo-EM particles. (C) Flowchart of the cryo-EM data processing. (D) Resolution estimation of the EM map. Gold standard Fourier shell correlation (FSC) curve, showing the overall nominal resolutions of 2.6 Å (E) Angular distributions of the cryo-EM particles in the final round of refinement. (F) local resolution map. A color scale at the bottom of each local resolution map indicates resolution (2.5-4 Å).
(DOCX)

S5 Fig Cryo-EM analysis of GX2012 spike trimers. (A) A representative cryo-EM micrograph from 1,288 micrographs. (B) 2D class averages of characteristic projection views of cryo-EM particles. (C) Flowchart of the cryo-EM data processing. (D) Resolution estimation of the EM map. Gold standard Fourier shell correlation (FSC) curve, showing the overall nominal resolutions of 3.0 Å (E) Angular distributions of the cryo-EM particles in the final round of refinement. (F) local resolution map. A color scale at the bottom of each local resolution map indicates resolution (2.5-4 Å).
(DOCX)

S6 Fig Structural comparison with MERS-CoV S glycoprotein. (A) Structure alignments between GX2012 and MERS-CoV S segments. GX2012 S segments were colored blue, RBM was colored red, and MERS-CoV S segments were colored magenta. As 2014-422 and GX2012 spikes have similar overall structures, thus only the GX2012 segments were used to show structural comparison with MERS-CoV. (B) Quantitative comparison between the 2014-422, GX2012 and MERS-CoV S proteins. The RMSD value is calculated using PyMOL. PDB code: MERS-CoV, 8IFN.
(DOCX)

S7 Fig The representative micrographs and 2D classification results of negative-staining EM. 2014-422 and GX2012 S proteins were incubated with hDPP4, and then inspected under negative-staining EM.
(DOCX)

S8 Fig Cryo-EM analysis of 2014-422 RBD-hDPP4 complex. (A) A representative cryo-EM micrograph from 4,095 micrographs. (B) 2D class averages of characteristic projection views of cryo-EM particles. (C) Flowchart of the cryo-EM data processing. (D) Resolution estimation of the EM maps. Gold standard Fourier shell correlation (FSC) curves, showing the overall nominal resolutions of 3.0 Å, 3.8 Å for the overall complex and 2014–422 RBD, respectively. (E) Angular distributions of the cryo-EM particles in the final round of refinement. (F) Local resolution map. A color scale at the bottom of each local resolution map indicates resolution (2.6-5.0 Å) for the overall complex and resolution (4.0-8.0 Å) for 2014-422 RBD.
(DOCX)

S9 Fig Cryo-EM analysis of GX2012 RBD-hDPP4 complex. (A) A representative cryo-EM micrograph from 1,827 micrographs. (B) 2D class averages of characteristic projection views of cryo-EM particles. (C) Flowchart of the cryo-EM data processing. (D) Resolution estimation of the EM maps. Gold standard Fourier shell correlation (FSC) curves, showing the overall nominal resolutions of 2.4 Å, 2.9 Å for the overall complex and GX2012 RBD, respectively. (E) Angular distributions of the cryo-EM particles in the final round of refinement. (F) Local resolution map. A color scale at the bottom of each local resolution map indicates resolution (2.2-4.6 Å) for the overall complex and resolution (2.6-5.0 Å) for GX2012 RBD.
(DOCX)

S10 Fig Overall structures of the 2014-422 and GX2012 RBD-hDPP4 complex. Overall structures of 2014-422 and GX2012 RBD-hDPP4 complex are shown in side views, with cryo-EM maps presented as semitransparent surfaces. 2014-422 RBD is in green, GX2012 RBD is in blue and hDPP4 is in dark grey.
(DOCX)

S11 Fig Hydrophobic interactions between 2014-422, GX2012, and MERS-CoV RBDs with hDPP4. The 2014-422, GX2012, and MERS-CoV RBDs are shown as surfaces according to hydrophobicity (green: hydrophilic; white: neutral; gold: hydrophobic). The helix from hDPP4 is shown in cartoon and sticks and colored dark grey.
(DOCX)

S12 Fig Biochemical and functional characterization of residue 514 mutants. (A) SDS-PAGE analysis of RBDs and hDPP4 proteins. (B) Binding curves of immobilized hDPP4 with 2014-422 RBD and 2014-422-d514 RBD. Data are shown as red dots, and the best fit of the data to a 1:1 binding model is shown as black lines. (C) The cell entry efficiency of 514 mutant pseudoviruses as measured by luciferase activity. Data are presented as mean values ± SD of five replicates.
(DOCX)

S13 Fig Structure comparison of 2014-422 RBD-hDPP4 and 2014-422-d514 RBD-hDPP4. (A) Predicted structure of 2014-422-d514 RBD-hDPP4 complex is shown in cartoon (slate blue: RBD; dark grey: hDPP4). The AlphaFold3 PAE plot for the model is shown in the right panel. (B) Structure alignments of 2014-422 RBD and 2014-422-d514 RBD based on hDPP4 (green: 2014-422 RBD; slate blue: 2014-422-d514 RBD; dark grey: hDPP4). N514 residue is shown in sticks. (C) Interfacing details of 2014-422/2014-422-d514 RBD-hDPP4 complexes.
(DOCX)

S14 Fig Structural comparison of two 2014-422 S trimers. (A) The two 2014-422 S trimers are aligned by the S2 subunit. The S trimer in this paper is in green, the S trimer from PDB:8SAK is in slate blue, and the JC57-11 antibody from PDB:8SAK is in salmon. (B) The RBD trimers of two 2014-422 are shown in the top view.
(DOCX)

S15 Fig Structural comparison of HKU4r RBDs-hDPP4 complex. (A) The binding interface of HKU4 RBD (PDB:4QZV), MjHKU4r RBD (PDB:8ZDZ), and GX2012 RBD. Three RBDs are shown as surfaces and colored orange, salmon and blue, respectively. The AA sequences of HKU4 and MjHKU4r RBD are renumbered based on the GX2012 RBD for convenient structural comparison. (B) Structure-based sequence alignment of the three RBDs with MERS-CoV. The external subdomain is highlighted by a red box. The binding epitope of GX2012, MjHKU4r, HKU4 and MERS-CoV-hDPP4 are labeled as blue angle, brown rectangle, orange circle and magenta rectangle, respectively.
(DOCX)

S1 Table Cryo-EM data collection, refinement, and validation statistics. (DOCX)

S2 Table Interfacing details of RBD-hDPP4 complexes. (DOCX)

S3 Table List of contact residues between RBD and hDPP4. (DOCX)
