# Supplementary material for: Structural basis for human DPP4 receptor recognition by MERS-like coronaviruses 2014-422 and GX2012
Source: PLoS Pathog. 2026 Jan 7;22(1):e1013792. doi: 10.1371/journal.ppat.1013792 (PMC12810913; doi:10.1371/journal.ppat.1013792)
Supplement: S1 Fig — SPR sensorgrams of immobilized hDPP4 binding to MERS-CoV (A), 2014-422 (B) and GX2012 (C) RBDs. Data are shown as colored lines. (DOCX) [file ppat.1013792.s001.docx]

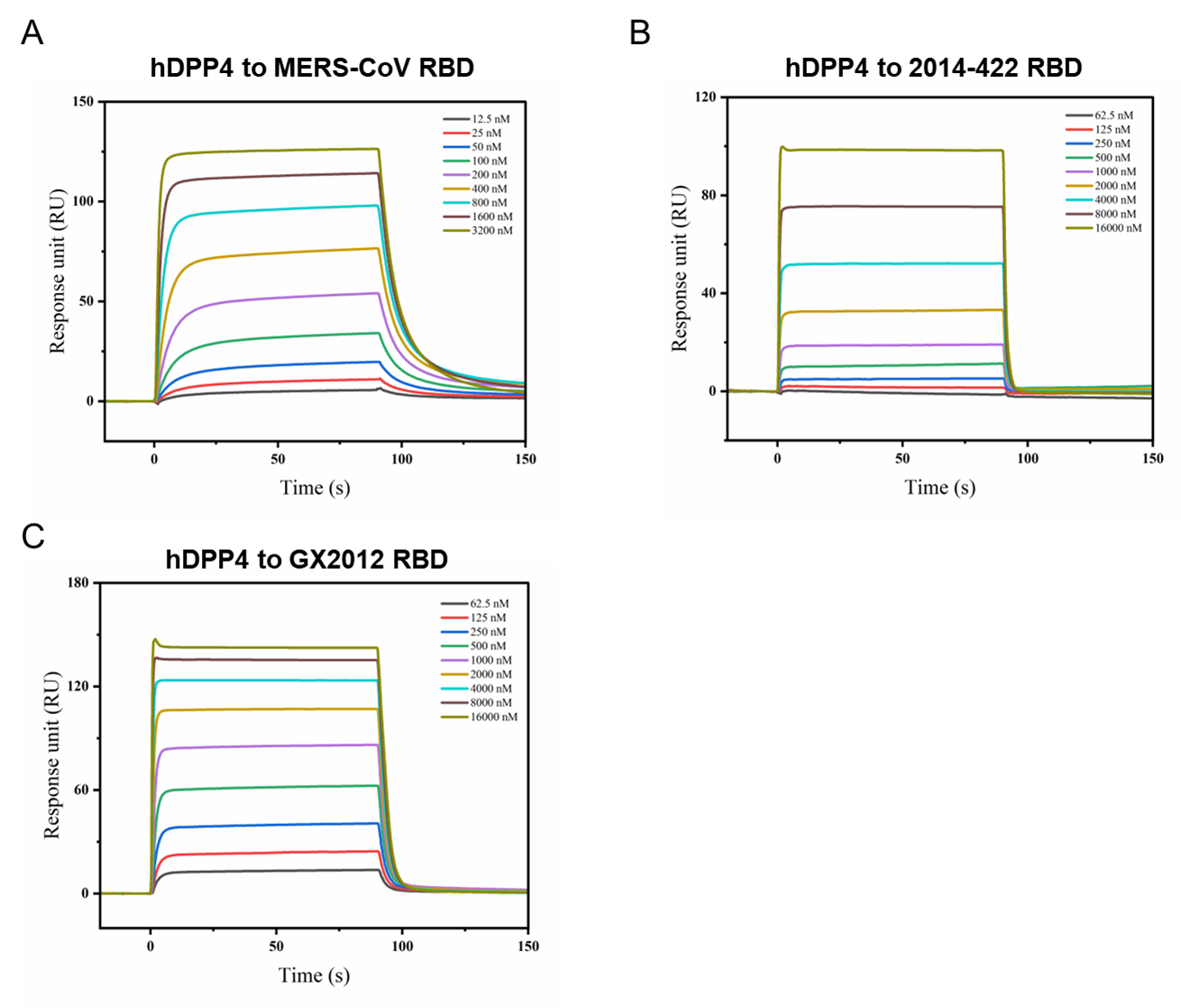


**S1 Fig SPR sensorgrams of hDPP4 with RBDs.** SPR sensorgrams of immobilized hDPP4 binding to MERS-CoV **(A)**, 2014-422 **(B)** and GX2012 **(C)** RBDs. Data are shown as colored lines.
