# Supplementary material for: Structural basis for human DPP4 receptor recognition by MERS-like coronaviruses 2014-422 and GX2012
Source: PLoS Pathog. 2026 Jan 7;22(1):e1013792. doi: 10.1371/journal.ppat.1013792 (PMC12810913; doi:10.1371/journal.ppat.1013792)
Supplement: S2 Fig — (A) Western blot analysis of MERS-CoV, 2014-422 and GX2012 spike pseudotype incorporation. All spike proteins tagged with C-terminal Strep-tag. The sample transfected with the pcDNA3.1 empty vector was used as a negative control. p24 used as the loading control. (B) Western blot analysis of MERS-CoV, 2014-422 and GX2012 spike with 15 µg/mL trypsin treatment pseudotype incorporation. (C) Western blot analysis of Huh-7 cells transfected with TMPRSS2 and Huh-7 WT cells. TMPRSS2 protein tagged with C-terminal Flag-tag. β-actin used as the loading control. (D) Western blot analysis of Spike mutants pseudotype incorporation. All spike proteins tagged with C-terminal Strep-tag. p24 used as the loading control. (DOCX) [file ppat.1013792.s002.docx]

**
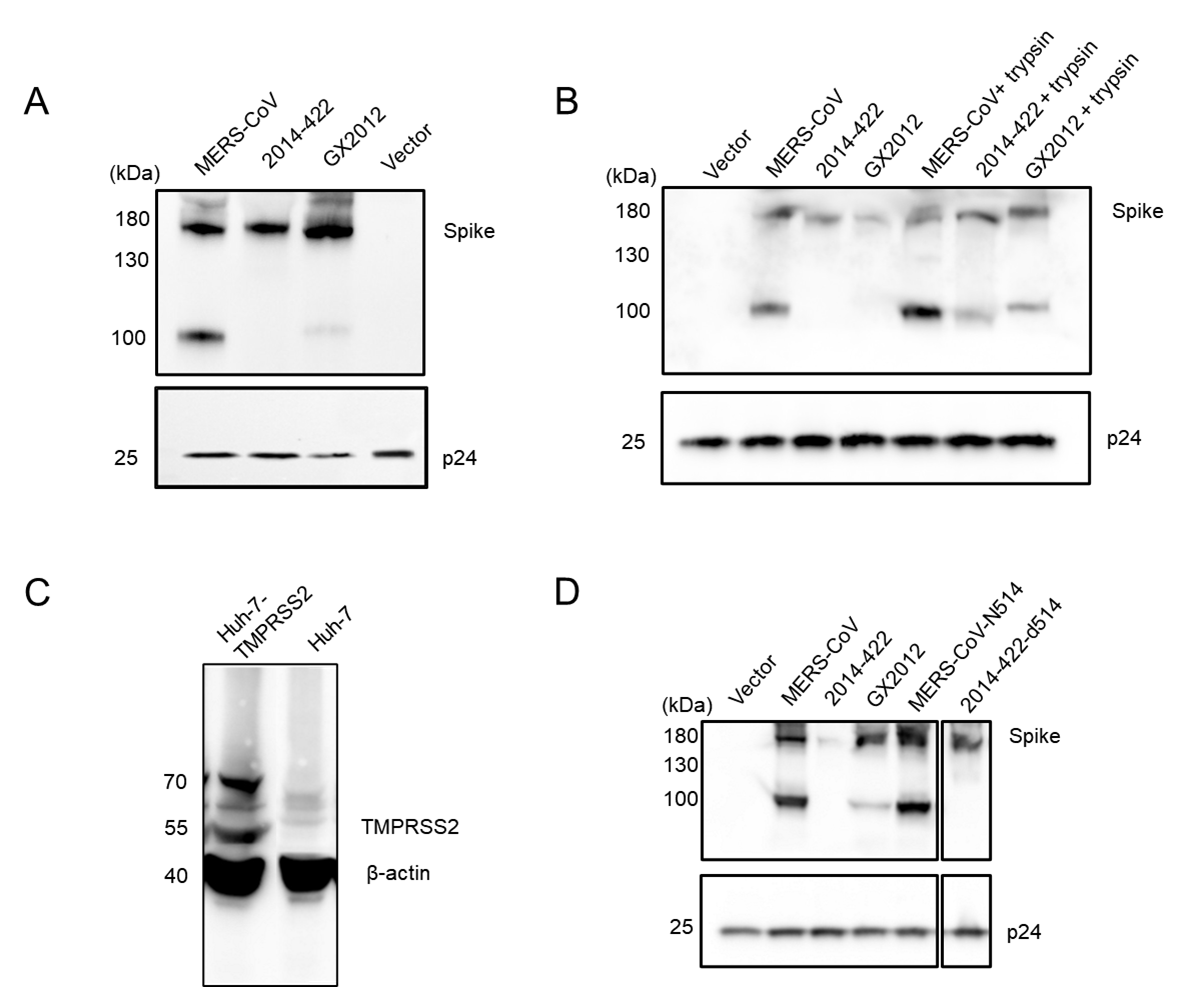
**

**S2 Fig Western blot analyses of spike pseudovirus incorporation and TMPRSS2 expression.** **(A)** Western blot analysis of MERS-CoV, 2014-422 and GX2012 spike pseudotype incorporation. All spike proteins tagged with C-terminal Strep-tag. The sample transfected with the pcDNA3.1 empty vector was used as a negative control. p24 used as the loading control. **(B)** Western blot analysis of MERS-CoV, 2014-422 and GX2012 spike with 15 µg/mL trypsin treatment pseudotype incorporation. **(C)** Western blot analysis of Huh-7 cells transfected with TMPRSS2 and Huh-7 WT cells. TMPRSS2 protein tagged with C-terminal Flag-tag. β-actin used as the loading control. **(D)** Western blot analysis of Spike mutants pseudotype incorporation. All spike proteins tagged with C-terminal Strep-tag. p24 used as the loading control.
