# Supplementary material for: Structural basis for human DPP4 receptor recognition by MERS-like coronaviruses 2014-422 and GX2012
Source: PLoS Pathog. 2026 Jan 7;22(1):e1013792. doi: 10.1371/journal.ppat.1013792 (PMC12810913; doi:10.1371/journal.ppat.1013792)
Supplement: S3 Fig — Size-exclusion chromatography profile and SDS-PAGE analysis of the gel-filtration elution. (A) 2014-422 Spike trimer. (B) GX2012 Spike trimer. (DOCX) [file ppat.1013792.s003.docx]

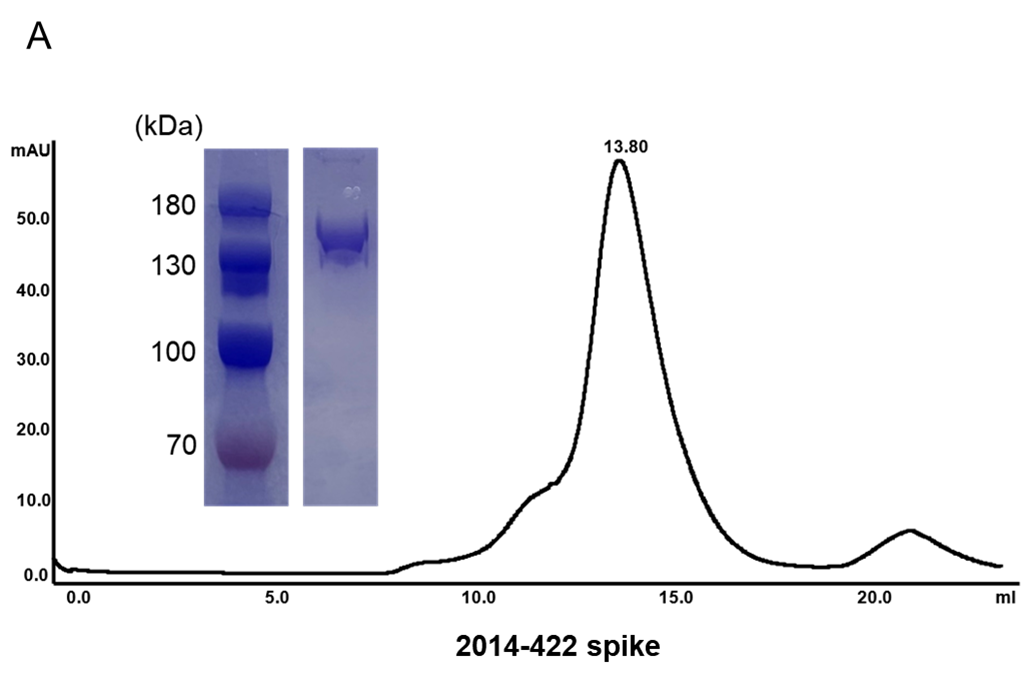


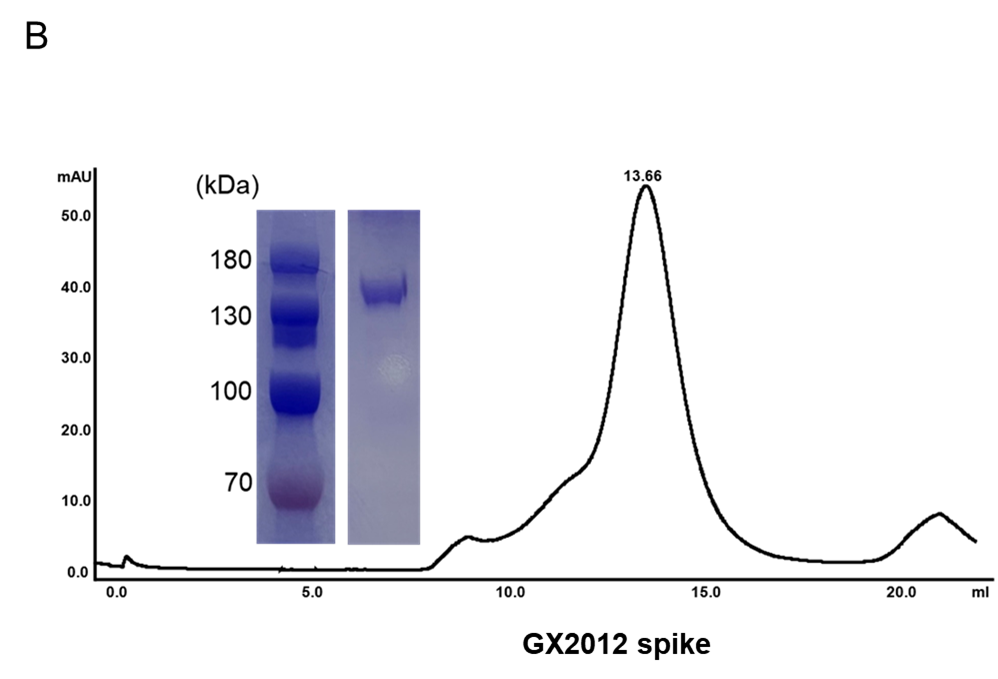


**S3 Fig Expression and purification of 2014-422 and GX2012 spike proteins.** Size-exclusion chromatography profile and SDS-PAGE analysis of the gel-filtration elution. **(A)** 2014-422 spike trimer. **(B)** GX2012 spike trimer.
