# Supplementary material for: Structural basis for human DPP4 receptor recognition by MERS-like coronaviruses 2014-422 and GX2012
Source: PLoS Pathog. 2026 Jan 7;22(1):e1013792. doi: 10.1371/journal.ppat.1013792 (PMC12810913; doi:10.1371/journal.ppat.1013792)
Supplement: S5 Fig — (A) A representative cryo-EM micrograph from 1,288 micrographs. (B) 2D class averages of characteristic projection views of cryo-EM particles. (C) Flowchart of the cryo-EM data processing. (D) Resolution estimation of the EM map. Gold standard Fourier shell correlation (FSC) curve, showing the overall nominal resolutions of 3.0 Å (E) Angular distributions of the cryo-EM particles in the final round of refinement. (F) local resolution map. A color scale at the bottom of each local resolution map indicates resolution (2.5-4 Å). (DOCX) [file ppat.1013792.s005.docx]

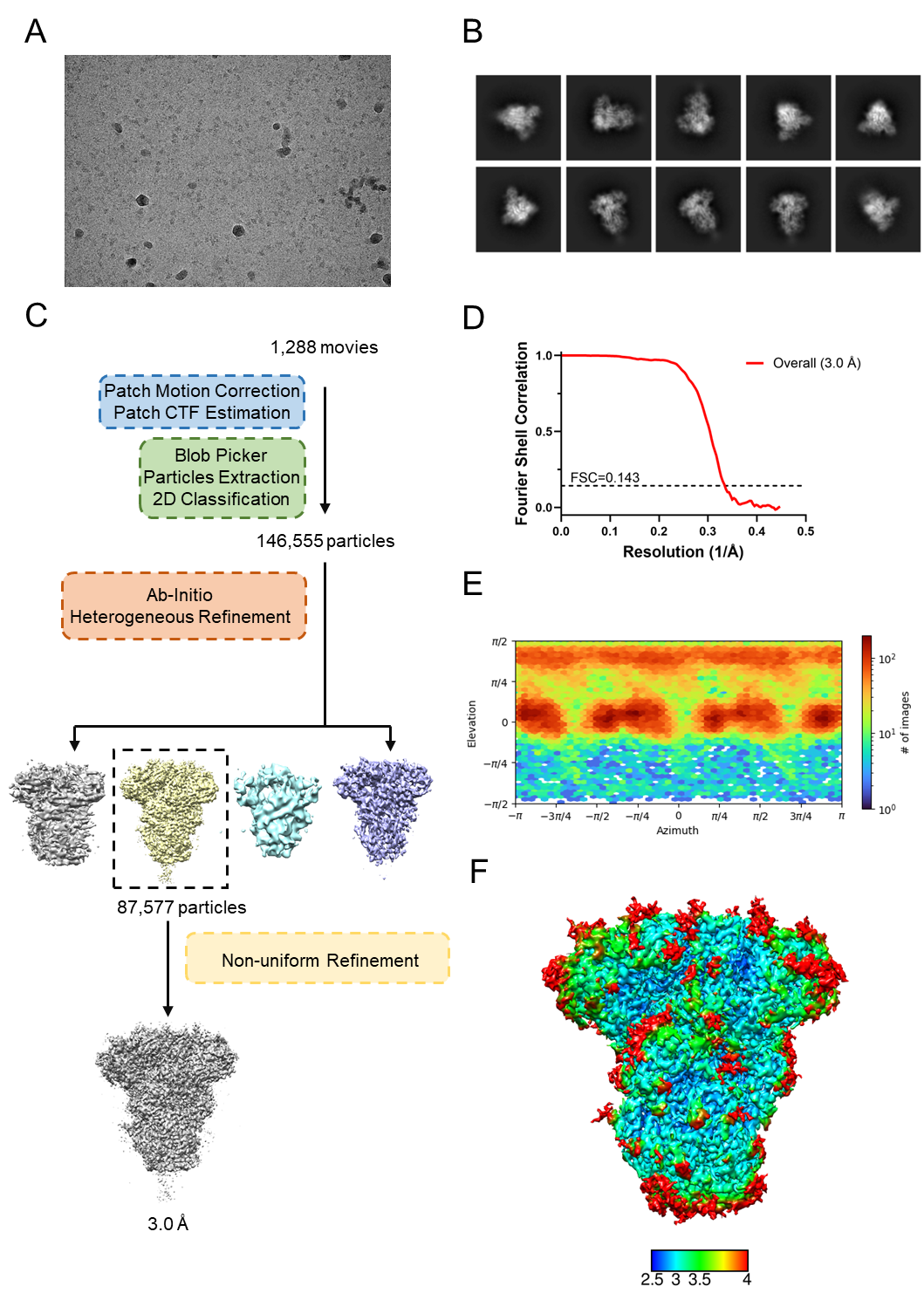


**S5 Fig Cryo-EM analysis of GX2012 spike trimers. (A)** A representative cryo-EM micrograph from 1,288 micrographs. **(B)** 2D class averages of characteristic projection views of cryo-EM particles. **(C)** Flowchart of the cryo-EM data processing. **(D)** Resolution estimation of the EM map. Gold standard Fourier shell correlation (FSC) curve, showing the overall nominal resolutions of 3.0 Å **(E)** Angular distributions of the cryo-EM particles in the final round of refinement. **(F)** local resolution map. A color scale at the bottom of each local resolution map indicates resolution (2.5-4 Å).
