# Supplementary material for: Structural basis for human DPP4 receptor recognition by MERS-like coronaviruses 2014-422 and GX2012
Source: PLoS Pathog. 2026 Jan 7;22(1):e1013792. doi: 10.1371/journal.ppat.1013792 (PMC12810913; doi:10.1371/journal.ppat.1013792)
Supplement: S6 Fig — (A) Structure alignments between GX2012 and MERS-CoV S segments. GX2012 S segments were colored blue, RBM was colored red, and MERS-CoV S segments were colored magenta. As 2014-422 and GX2012 spikes have similar overall structures, thus only the GX2012 segments were used to show structural comparison with MERS-CoV. (B) Quantitative comparison between the 2014-422, GX2012 and MERS-CoV S proteins. The RMSD value is calculated using PyMOL. PDB code: MERS-CoV, 8IFN. (DOCX) [file ppat.1013792.s006.docx]

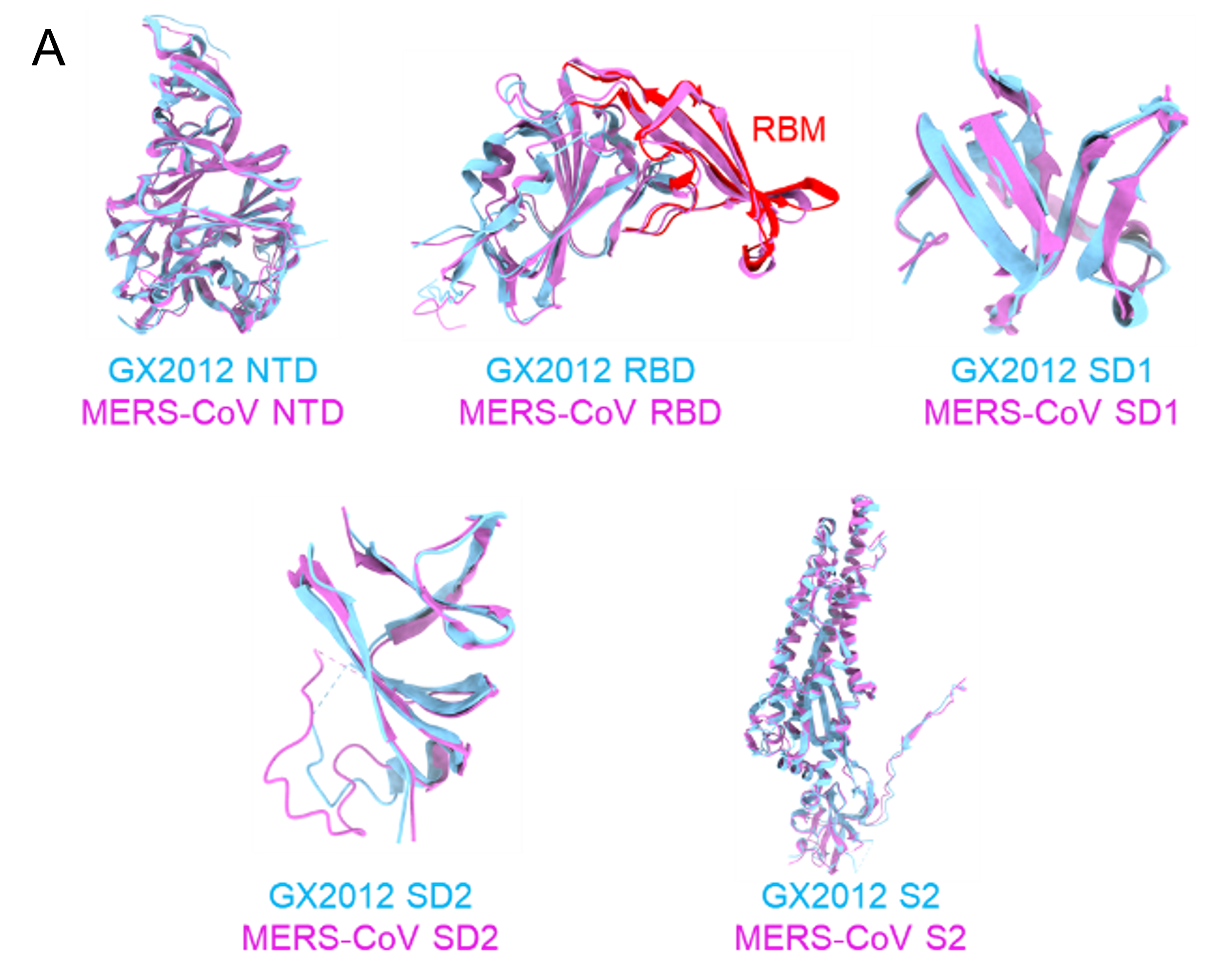


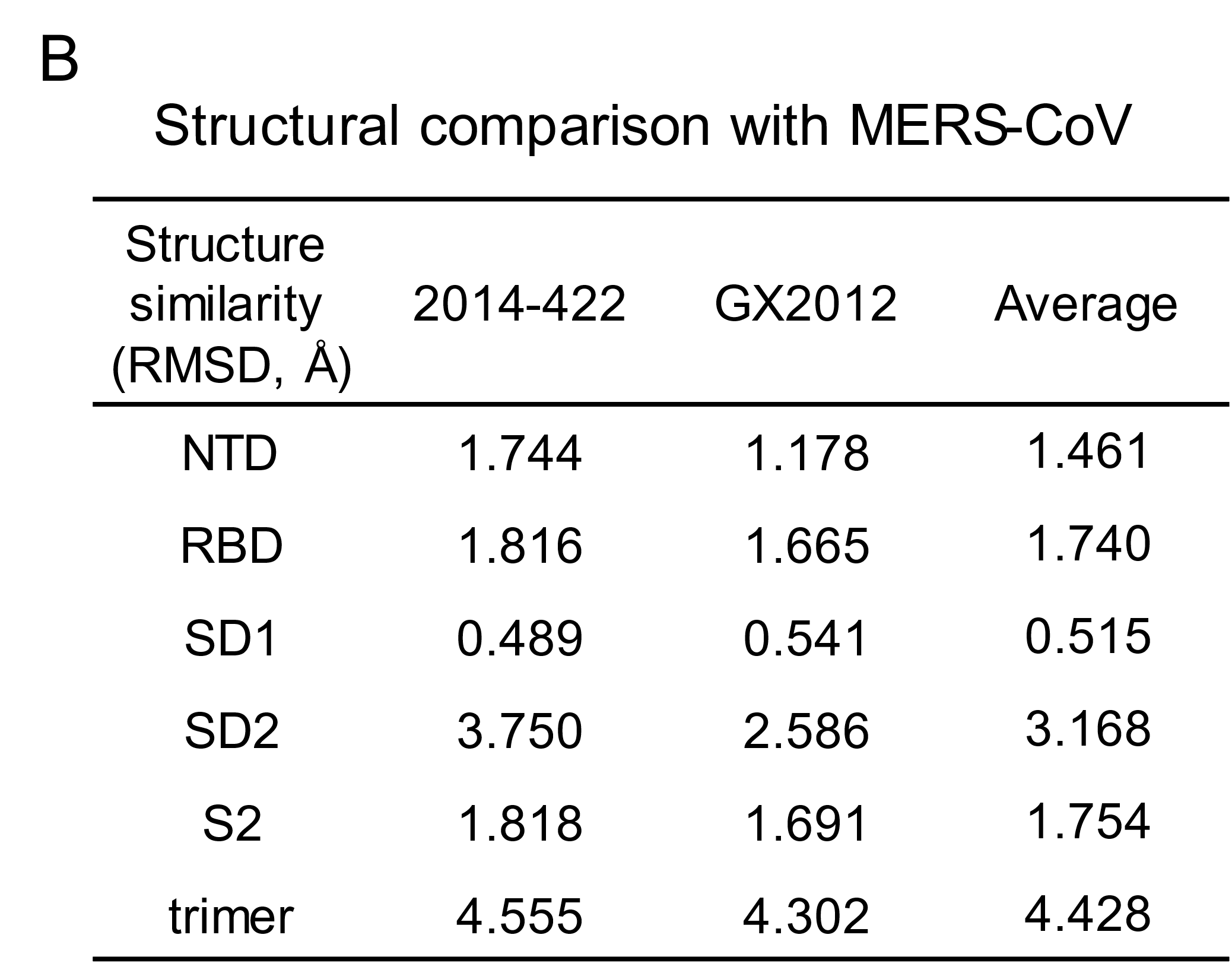


**S6 Fig Structural comparison with MERS-CoV spike glycoprotein. (A)** Structure alignments between GX2012 and MERS-CoV spike segments. GX2012 spike segments were colored blue, RBM was colored red, and MERS-CoV spike segments were colored magenta. As 2014-422 and GX2012 spikes have similar overall structures, thus only the GX2012 segments were used to show structural comparison with MERS-CoV. **(B)** Quantitative comparison between the 2014-422, GX2012 and MERS-CoV spike proteins. The RMSD value is calculated using PyMOL. PDB code: MERS-CoV, 8IFN.
