# Supplementary material for: Structural basis for human DPP4 receptor recognition by MERS-like coronaviruses 2014-422 and GX2012
Source: PLoS Pathog. 2026 Jan 7;22(1):e1013792. doi: 10.1371/journal.ppat.1013792 (PMC12810913; doi:10.1371/journal.ppat.1013792)
Supplement: S7 Fig — 2014-422 and GX2012 S proteins were incubated with hDPP4, and then inspected under negative-staining EM. (DOCX) [file ppat.1013792.s007.docx]

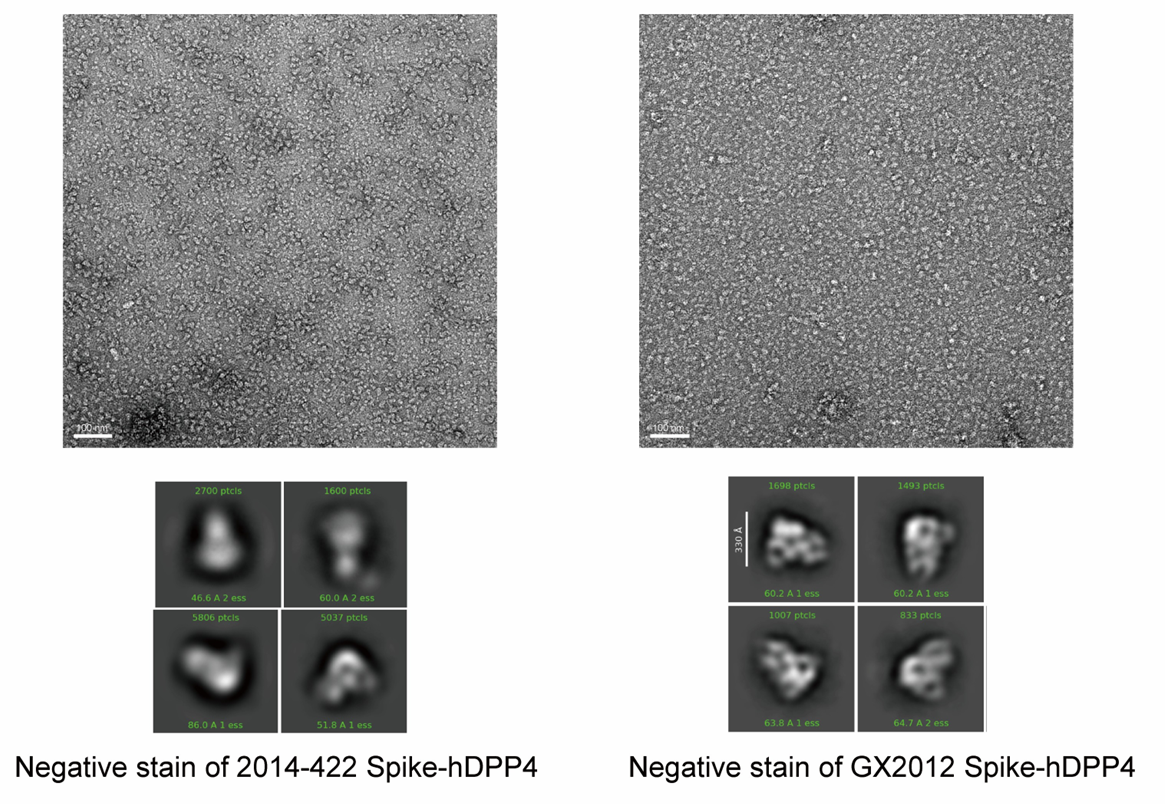


**S7 Fig The representative micrographs and 2D classification results of negative-staining EM.** 2014-422 and GX2012 spike proteins were incubated with hDPP4, and then inspected under negative-staining EM.
