# Supplementary material for: Structural basis for human DPP4 receptor recognition by MERS-like coronaviruses 2014-422 and GX2012
Source: PLoS Pathog. 2026 Jan 7;22(1):e1013792. doi: 10.1371/journal.ppat.1013792 (PMC12810913; doi:10.1371/journal.ppat.1013792)
Supplement: S10 Fig — Overall structures of 2014-422 and GX2012 RBD-hDPP4 complex are shown in side views, with cryo-EM maps presented as semitransparent surfaces. 2014-422 RBD is in green, GX2012 RBD is in blue and hDPP4 is in dark grey. (DOCX) [file ppat.1013792.s010.docx]

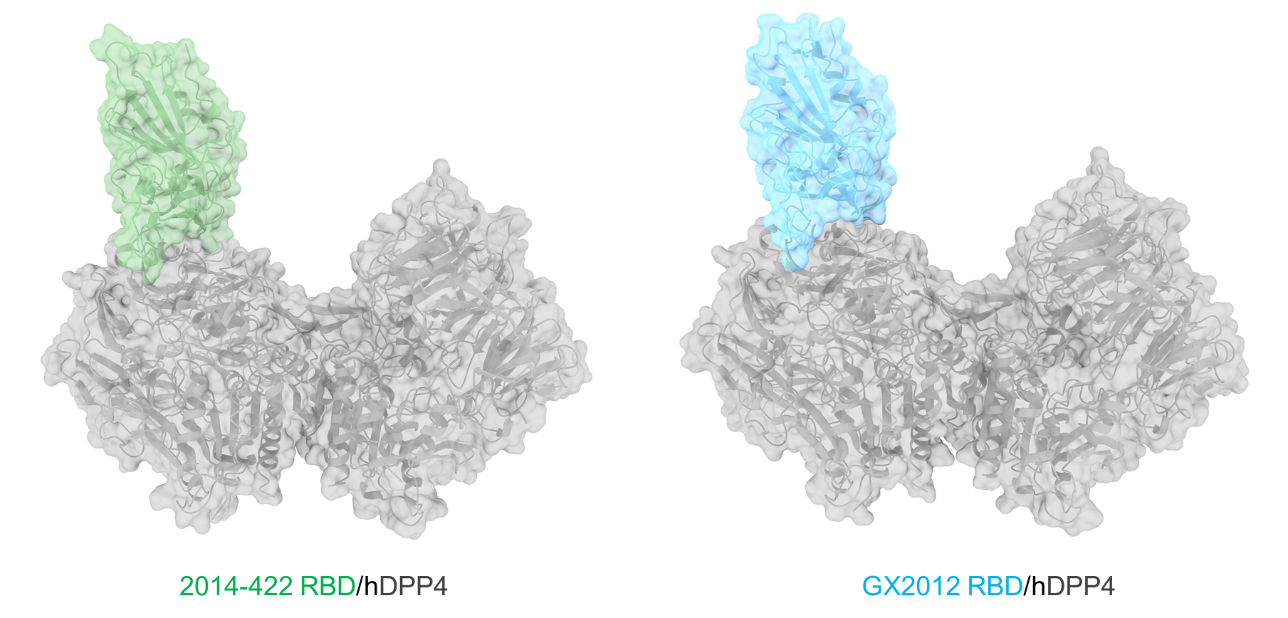


**S10 Fig Overall structures of the 2014-422 and GX2012 RBD-hDPP4 complex.** Overall structures of 2014-422 and GX2012 RBD-hDPP4 complex are shown in side views, with cryo-EM maps presented as semitransparent surfaces. 2014-422 RBD is in green, GX2012 RBD is in blue and hDPP4 is in dark grey.
