# Supplementary material for: Structural basis for human DPP4 receptor recognition by MERS-like coronaviruses 2014-422 and GX2012
Source: PLoS Pathog. 2026 Jan 7;22(1):e1013792. doi: 10.1371/journal.ppat.1013792 (PMC12810913; doi:10.1371/journal.ppat.1013792)
Supplement: S11 Fig — The 2014-422, GX2012, and MERS-CoV RBDs are shown as surfaces according to hydrophobicity (green: hydrophilic; white: neutral; gold: hydrophobic). The helix from hDPP4 is shown in cartoon and sticks and colored dark grey. (DOCX) [file ppat.1013792.s011.docx]

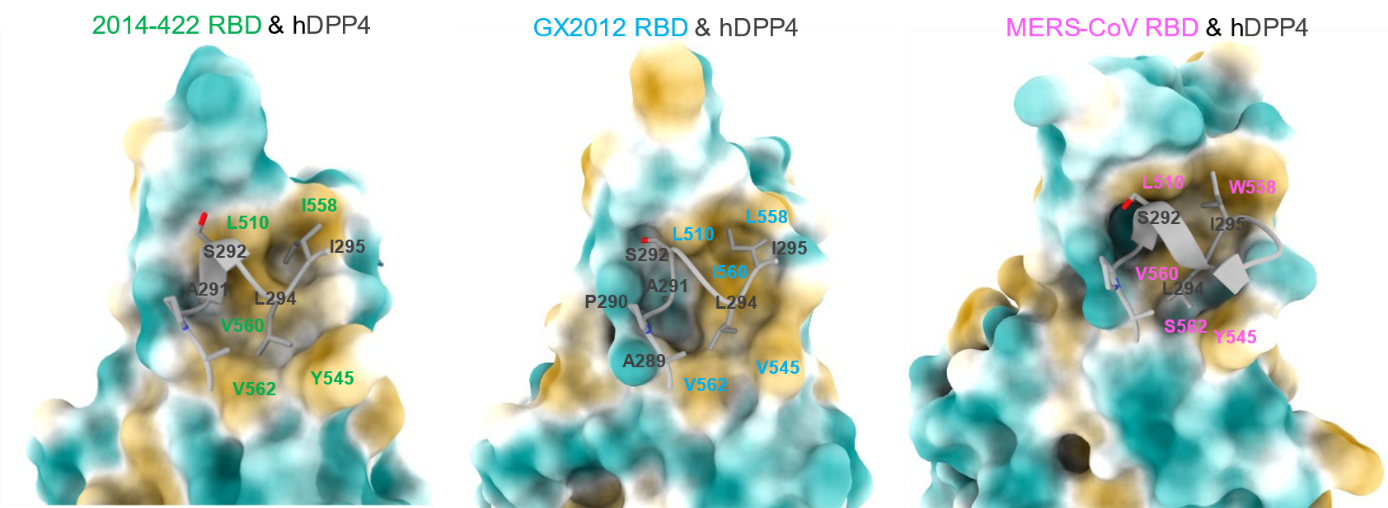


**S11 Fig Hydrophobic interactions between the 2014-422, GX2012, and MERS-CoV RBDs with hDPP4.** The 2014-422, GX2012, and MERS-CoV RBDs are shown as surfaces according to hydrophobicity (green: hydrophilic; white: neutral; gold: hydrophobic). The helix from hDPP4 is shown in cartoon and sticks and colored dark grey.
