# Supplementary material for: Structural basis for human DPP4 receptor recognition by MERS-like coronaviruses 2014-422 and GX2012
Source: PLoS Pathog. 2026 Jan 7;22(1):e1013792. doi: 10.1371/journal.ppat.1013792 (PMC12810913; doi:10.1371/journal.ppat.1013792)
Supplement: S12 Fig — (A) SDS-PAGE analysis of RBDs and hDPP4 proteins. (B) Binding curves of immobilized hDPP4 with 2014-422 RBD and 2014-422-d514 RBD. Data are shown as red dots, and the best fit of the data to a 1:1 binding model is shown as black lines. (C) The cell entry efficiency of 514 mutant pseudoviruses as measured by luciferase activity. Data are presented as mean values ± SD of five replicates. (DOCX) [file ppat.1013792.s012.docx]

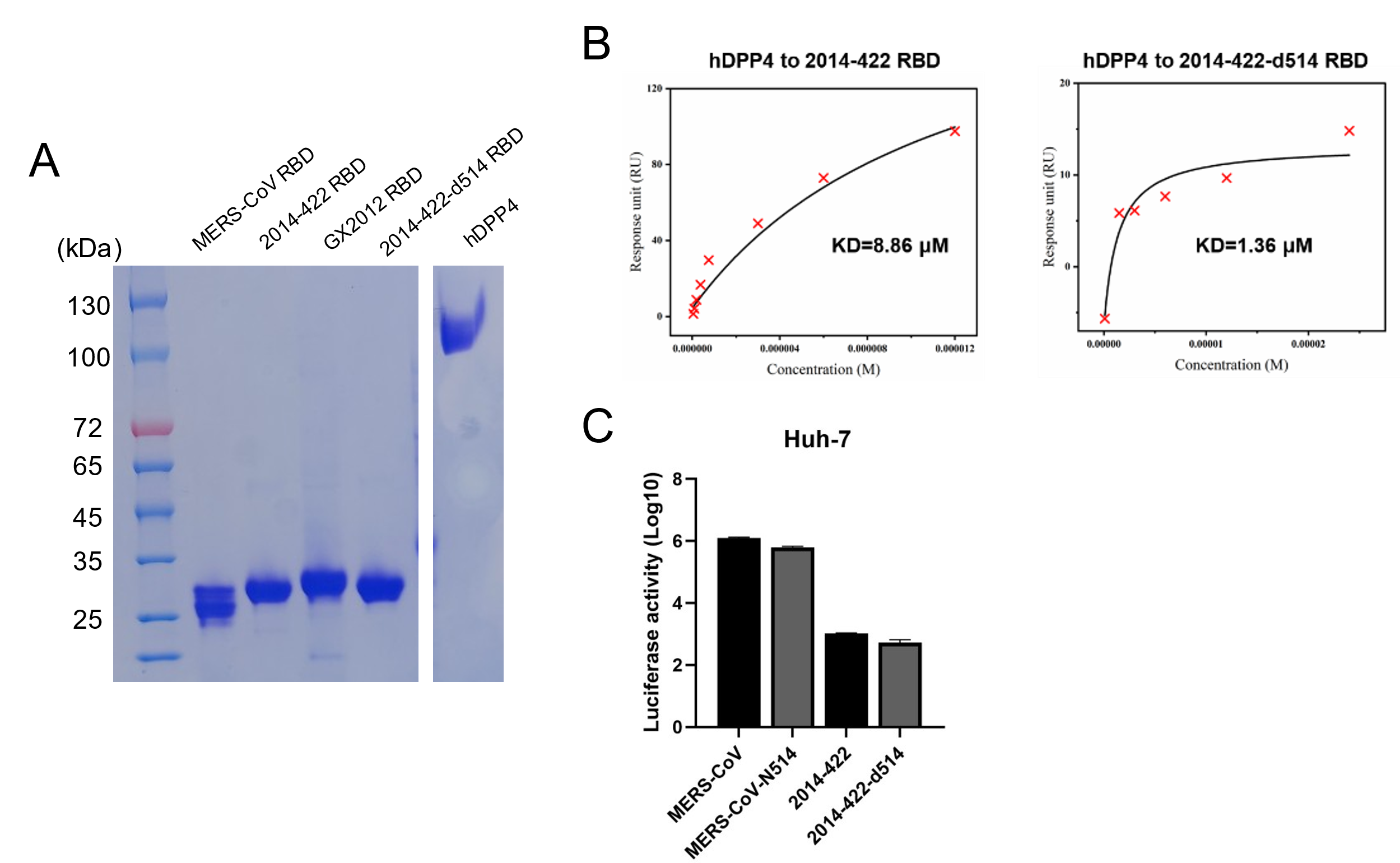


**S12 Fig Biochemical and functional characterization of residue 514 mutants. (A)** SDS-PAGE analysis of RBDs and hDPP4 proteins. **(B)** Binding curves of immobilized hDPP4 with 2014-422 RBD and 2014-422-d514 RBD. Data are shown as red dots, and the best fit of the data to a 1:1 binding model is shown as black lines. **(C)** The cell entry efficiency of 514 mutant pseudoviruses as measured by luciferase activity. Data are presented as mean values ± SD of five replicates.
