# Supplementary material for: Structural basis for human DPP4 receptor recognition by MERS-like coronaviruses 2014-422 and GX2012
Source: PLoS Pathog. 2026 Jan 7;22(1):e1013792. doi: 10.1371/journal.ppat.1013792 (PMC12810913; doi:10.1371/journal.ppat.1013792)
Supplement: S13 Fig — (A) Predicted structure of 2014-422-d514 RBD-hDPP4 complex is shown in cartoon (slate blue: RBD; dark grey: hDPP4). The AlphaFold3 PAE plot for the model is shown in the right panel. (B) Structure alignments of 2014-422 RBD and 2014-422-d514 RBD based on hDPP4 (green: 2014-422 RBD; slate blue: 2014-422-d514 RBD; dark grey: hDPP4). N514 residue is shown in sticks. (C) Interfacing details of 2014-422/2014-422-d514 RBD-hDPP4 complexes. (DOCX) [file ppat.1013792.s013.docx]

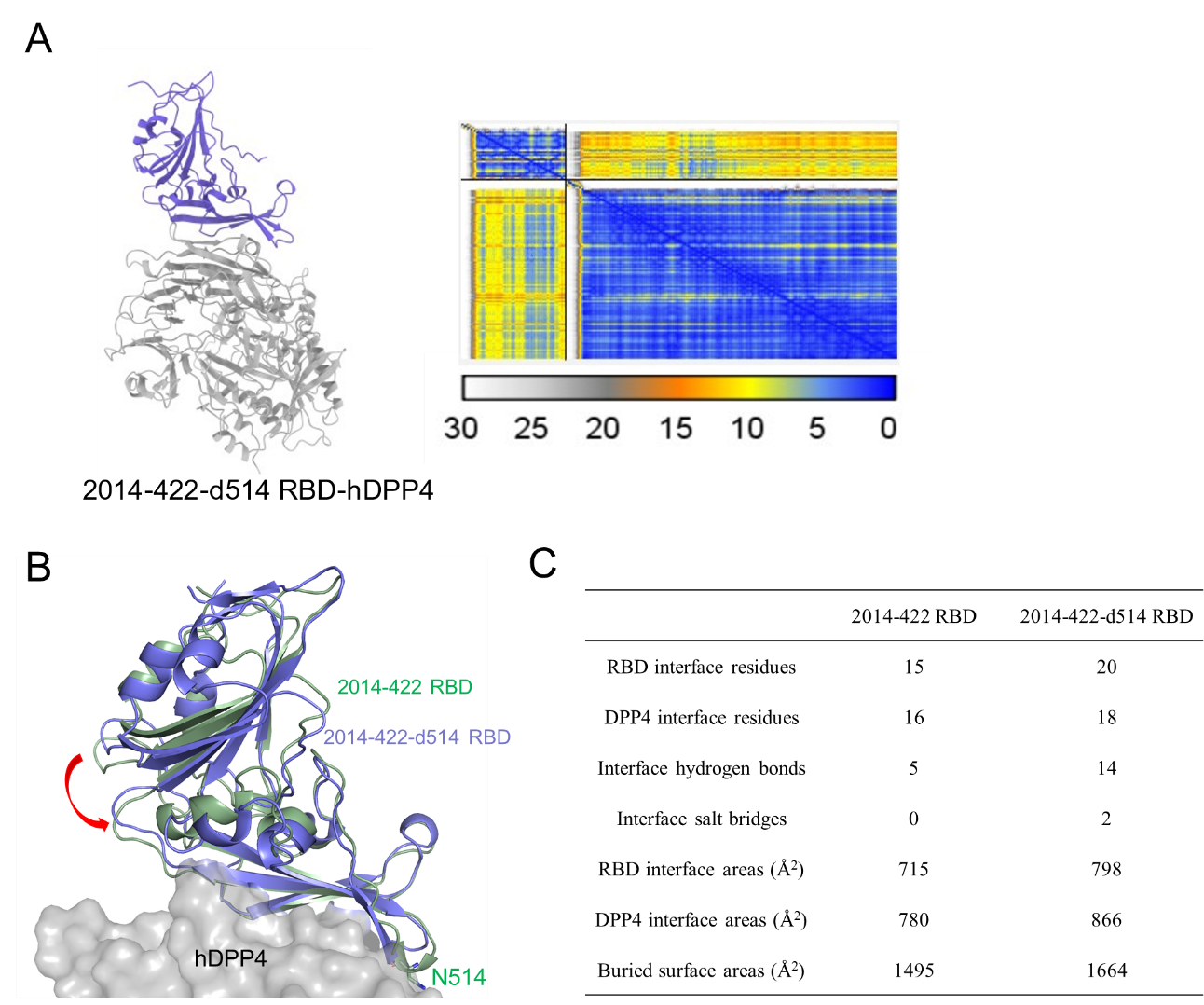


**S13 Fig Structure comparison of 2014-422 RBD-hDPP4 and 2014-422-d514 RBD-hDPP4. (A)** Predicted structure of 2014-422-d514 RBD-hDPP4 complex is shown in cartoon (slate blue: RBD; dark grey: hDPP4). The AlphaFold3 PAE plot for the model is shown in the right panel. **(B)** Structure alignments of 2014-422 RBD and 2014-422-d514 RBD based on hDPP4 (green: 2014-422 RBD; slate blue: 2014-422-d514 RBD; dark grey: hDPP4). N514 residue is shown in sticks. **(C)** Interfacing details of 2014-422/2014-422-d514 RBD-hDPP4 complexes.
