# Supplementary material for: Structural basis for human DPP4 receptor recognition by MERS-like coronaviruses 2014-422 and GX2012
Source: PLoS Pathog. 2026 Jan 7;22(1):e1013792. doi: 10.1371/journal.ppat.1013792 (PMC12810913; doi:10.1371/journal.ppat.1013792)
Supplement: S14 Fig — (A) The two 2014-422 S trimers are aligned by the S2 subunit. The S trimer in this paper is in green, the S trimer from PDB:8SAK is in slate blue, and the JC57-11 antibody from PDB:8SAK is in salmon. (B) The RBD trimers of two 2014-422 are shown in the top view. (DOCX) [file ppat.1013792.s014.docx]

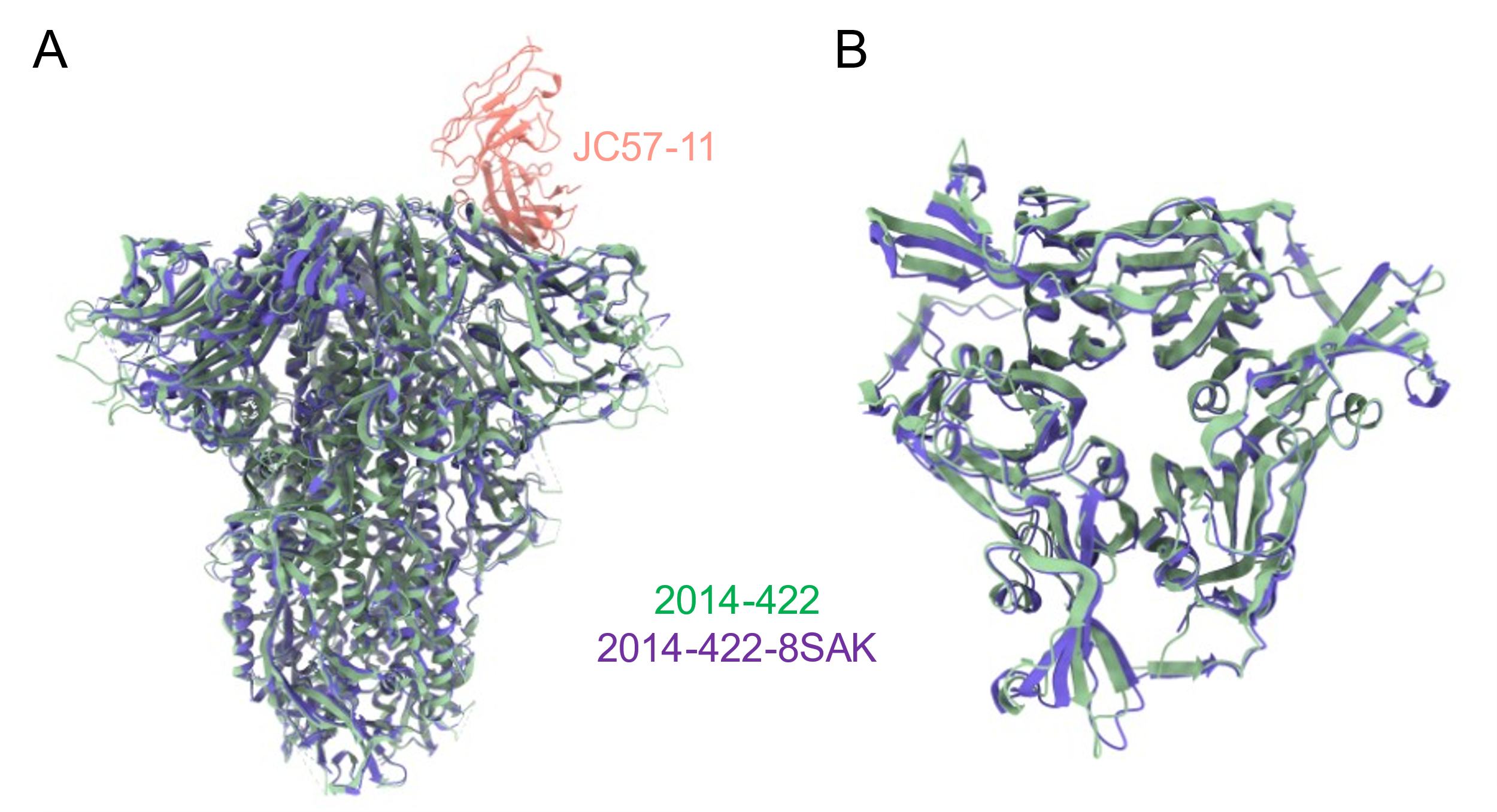


**S14 Fig. Structural comparison of two 2014-422 S trimers. (A)** The two 2014-422 S trimers are aligned by the S2 subunit. The S trimer in this paper is in green, the S trimer from PDB:8SAK is in slate blue, and the JC57-11 antibody from PDB:8SAK is in salmon. **(B)** The RBD trimers of two 2014-422 are shown in the top view.
