# Supplementary material for: Structural basis for human DPP4 receptor recognition by MERS-like coronaviruses 2014-422 and GX2012
Source: PLoS Pathog. 2026 Jan 7;22(1):e1013792. doi: 10.1371/journal.ppat.1013792 (PMC12810913; doi:10.1371/journal.ppat.1013792)
Supplement: S15 Fig — (A) The binding interface of HKU4 RBD (PDB:4QZV), MjHKU4r RBD (PDB:8ZDZ), and GX2012 RBD. Three RBDs are shown as surfaces and colored orange, salmon and blue, respectively. The AA sequences of HKU4 and MjHKU4r RBD are renumbered based on the GX2012 RBD for convenient structural comparison. (B) Structure-based sequence alignment of the three RBDs with MERS-CoV. The external subdomain is highlighted by a red box. The binding epitope of GX2012, MjHKU4r, HKU4 and MERS-CoV-hDPP4 are labeled as blue angle, brown rectangle, orange circle and magenta rectangle, respectively. (DOCX) [file ppat.1013792.s015.docx]

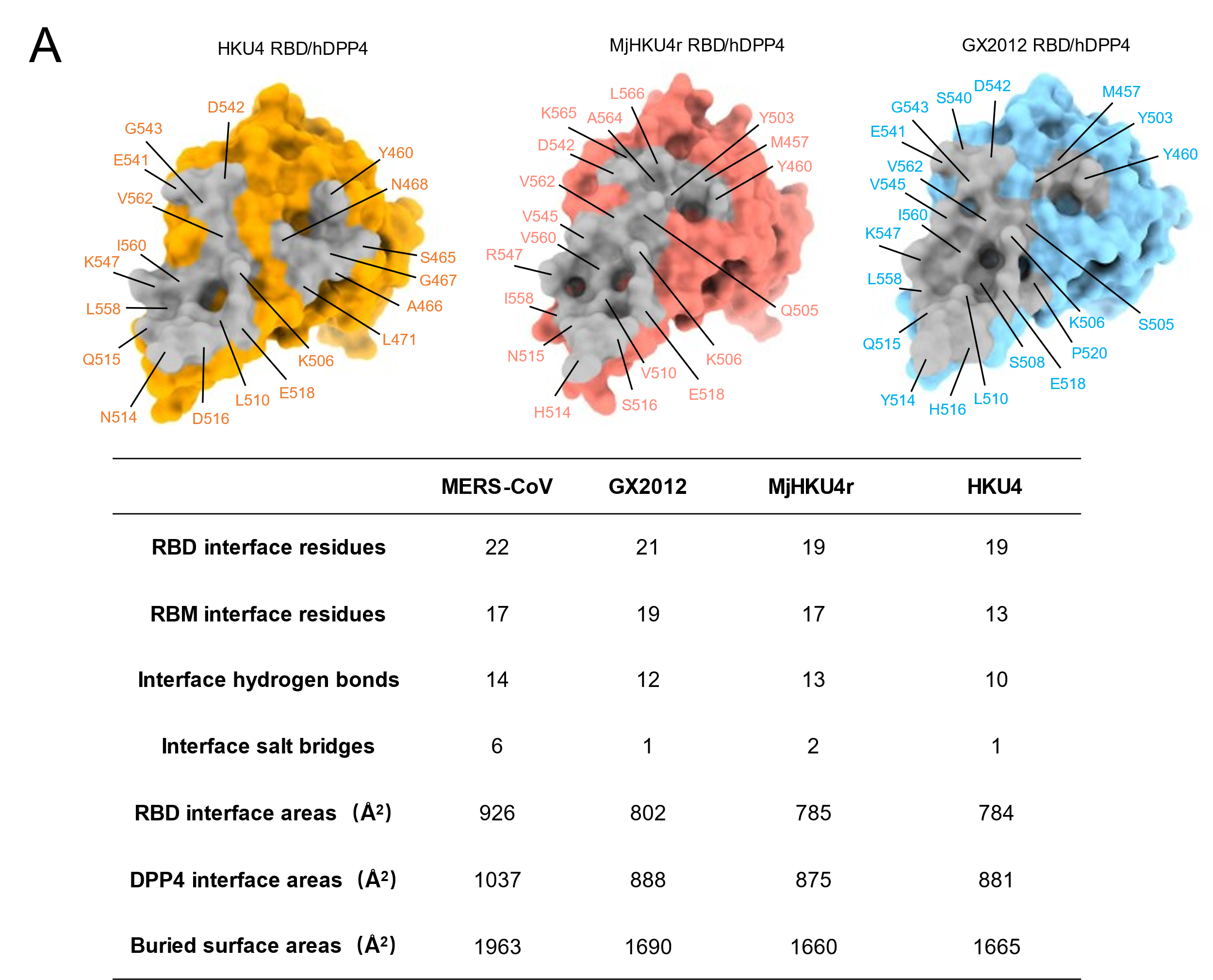


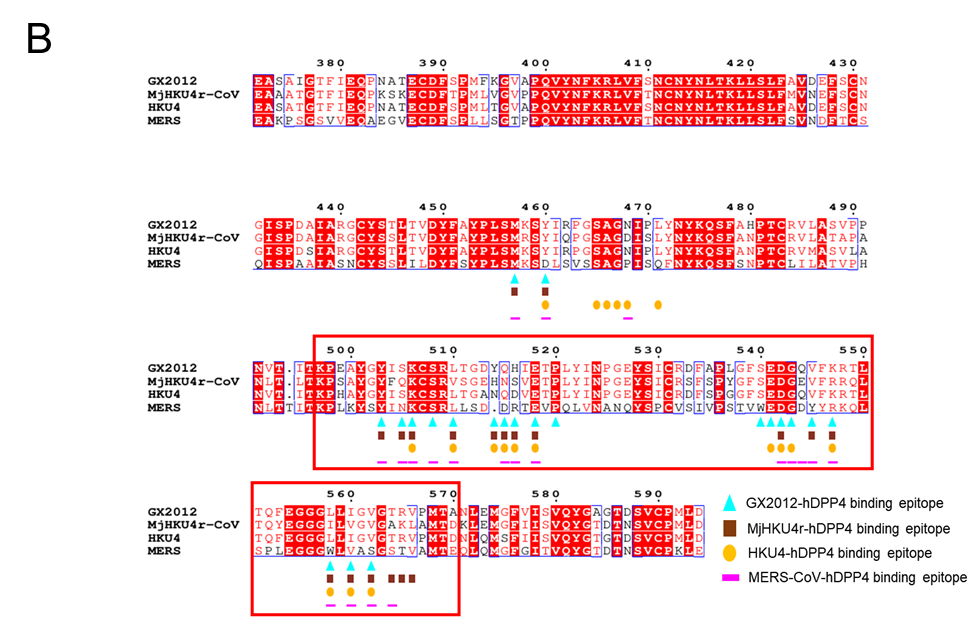


**S15 Fig Structural comparison of HKU4r RBDs-hDPP4 complex. (A)** The binding interface of HKU4 RBD (PDB:4QZV), MjHKU4r RBD (PDB:8ZDZ), and GX2012 RBD. Three RBDs are shown as surfaces and colored orange, salmon and blue, respectively. The AA sequences of HKU4 and MjHKU4r RBD are renumbered based on the GX2012 RBD for convenient structural comparison. **(B)** Structure-based sequence alignment of the three RBDs with MERS-CoV. The external subdomain is highlighted by a red box. The binding epitope of GX2012, MjHKU4r, HKU4 and MERS-CoV-hDPP4 are labeled as blue angle, brown rectangle, orange circle and magenta rectangle, respectively.
