# Supplementary material for: Structural basis for human DPP4 receptor recognition by MERS-like coronaviruses 2014-422 and GX2012
Source: PLoS Pathog. 2026 Jan 7;22(1):e1013792. doi: 10.1371/journal.ppat.1013792 (PMC12810913; doi:10.1371/journal.ppat.1013792)
Supplement: S1 Table — (DOCX) [file ppat.1013792.s016.docx]

**S1 Table Cryo-EM data collection, refinement, and validation statistics**

|  | 2014-422 spike | 22014-422 RBD-hDPP4  W | GX2012 spike | | | GX2012 RBD-hDPP4 |
| --- | --- | --- | --- | --- | --- | --- |
|  | (EMD-64080, PDB 9UEB) | (EMD-64732, PDB 9V2L) | | (EMD-64079, PDB 9UEA) | (EMD-64736, PDB 9V2P) | |
| **Data collection and processing** | | | | | | |
| Magnification | ×64000 | ×64000 | | ×64000 | ×81000 | |
| Voltage (kV) | 300 | 300 | | 300 | 300 | |
| Electron exposure (e^–^/Å^2^) | 50 | 50 | | 50 | 50 | |
| Defocus range (μm) | -1.5 to -1.8 | -1.5 to -1.8 | | -1.5 to -1.8 | -1.5 to -1.8 | |
| Pixel size (Å) | 1.0979 | 1.0979 | | 1.0979 | 1.0742 | |
| Symmetry imposed | C3 | C1 | | C3 | C1 | |
| Initial particle images (no.) | ~1,510,000 | ~2,628,000 | | ~1,308,000 | ~5,810,000 | |
| Final particle images (no.) | 346,468 | 591,107 | | 87,577 | 447,216 | |
| Map resolution (Å) | 2.59 | 3.0 | | 2.94 | 2.4 | |
| FSC threshold | 0.143 | 0.143 | | 0.143 | 0.143 | |
| Map resolution range (Å) | 2.5-4.0 | 2.6-5.0 | | 2.5-4.0 | 2.2-4.6 | |
| **Refinement** | | | | | | |
| Initial model used | CryoNet | CryoNet | | CryoNet | CryoNet | |
| Map sharpening  *B* factor (Å^2^) | -106.9 | -139.2 | | -110.7 | -77.1 | |
| **Model composition** |  |  | |  |  | |
| non-hydrogen atoms | 27524 | 13652 | | 27746 | 13891 | |
| Protein residues | 3518 | 1657 | | 3521 | 1663 | |
| Ligands | 30 | 13 | | 46 | 26 | |
| ***B* factors (Å^2^)** |  |  | |  |  | |
| Protein | 44.10 | 32.33 | | 62.45 | 6.19 | |
| Ligand | 64.37 | 51.32 | | 30 | 30 | |
| **R.m.s. deviations** |  |  | |  |  | |
| Bond lengths (Å) | 0.003 | 0.005 | | 0.004 | 0.007 | |
| Bond angles (°) | 0.642 | 0.756 | | 0.678 | 1.238 | |
| **Validation** |  |  | |  |  | |
| MolProbity score | 1.80 | 1.82 | | 1.66 | 2.16 | |
| Clashscore | 7.19 | 8.18 | | 6.13 | 10.91 | |
| Poor rotamers (%) | 0.00 | 0.27 | | 0.00 | 2.16 | |
| **Ramachandran plot** |  |  | |  |  | |
| Favored (%) | 93.94 | 94.49 | | 95.30 | 94.87 | |
| Allowed (%) | 6.00 | 5.45 | | 4.44 | 4.77 | |
| Disallowed (%) | 0.06 | 0.06 | | 0.26 | 0.36 | |
