# Supplementary material for: Structural basis for human DPP4 receptor recognition by MERS-like coronaviruses 2014-422 and GX2012
Source: PLoS Pathog. 2026 Jan 7;22(1):e1013792. doi: 10.1371/journal.ppat.1013792 (PMC12810913; doi:10.1371/journal.ppat.1013792)
Supplement: S2 Table — (DOCX) [file ppat.1013792.s017.docx]

**S2 Table Interfacing details of RBD-hDPP4 complexes**

|  | 2014-422 RBD | GX2012 RBD | MERS-CoV RBD |
| --- | --- | --- | --- |
| RBD interface residues | 15 | 21 | 22 |
| DPP4 interface residues | 16 | 17 | 19 |
| Interface hydrogen bonds | 5 | 12 | 14 |
| Interface salt bridges | 0 | 1 | 6 |
| RBD interface areas (Å^2^) | 715 | 802 | 926 |
| DPP4 interface areas (Å^2^) | 780 | 888 | 1037 |
| Buried surface areas (Å^2^) | 1495 | 1690 | 1963 |
