# Supplementary material for: Structural basis for human DPP4 receptor recognition by MERS-like coronaviruses 2014-422 and GX2012
Source: PLoS Pathog. 2026 Jan 7;22(1):e1013792. doi: 10.1371/journal.ppat.1013792 (PMC12810913; doi:10.1371/journal.ppat.1013792)
Supplement: S3 Table — (DOCX) [file ppat.1013792.s018.docx]

**S3 Table List of contact residues between RBD and hDPP4**

| hDPP4 | 2014-422 RBD | GX2012 RBD | MERS-CoV RBD |
| --- | --- | --- | --- |
| T265 |  |  | Y545 |
| V266 | Y545 |  |  |
| K267 | Q541 | E541, D542 | D542, G543, D544 |
| F269 |  |  | D542 |
| Q286 | D542, G543 | D542, G543 | N505, G543,  S562, S564 |
| T288 | G543 | S505, K506, V562 | N505, K506, S562 |
| A289 |  | K506 | K506 |
| P290 |  | K506 | E518 |
| A291 | K506, L510, E518 | S508, E518 | K506, S508, L510,  E518, V560 |
| S292 | L510, S515, E518 | L510, Q515,  H516, E518 | L510 |
| L294 | Y545, V560, V562 | V545, I560 | K506, Y545,  R547, V562 |
| I295 | S515, I558, V560 | L510, K547, L558 | L510, D515,  R547, W558 |
| G296 |  |  | R547 |
| H298 |  |  | Y545 |
| R317 | N514, S515 | Y514, Q515 | D515 |
| Y322 | H516 | H516 | D515, R516 |
| S334 | Y460 | Y460 |  |
| G335 |  | Y460 |  |
| R336 | F503 | M457, Y460, Y503 | M457, D460,  P468, Y503 |
| V341 | E518 | P520 | E518 |
| Q344 | E518 | E518 | E518 |
| I346 | H516 |  | R516 |
| M348 | N514 | Y514 |  |
| N229 glycan |  | S540, E541 | W540, E541 |
| N321 glycan | N514 | Y514 |  |
